# *Claroideglomus etunicatum* enhances *Pteris vittata* L. arsenic resistance and accumulation by mediating the rapid reduction and transport of arsenic in roots

**DOI:** 10.3389/fpls.2024.1464547

**Published:** 2024-11-13

**Authors:** Guofei Pan, YueZhen Xu, WeiZhen Li, Linyan Zan, Xueli Wang

**Affiliations:** Guangxi Key Laboratory for Agro-Environment and Agro-Products Safety, State Key Laboratory for Conservation and Utilization of Subtropical Agri–Bioresources, National Demonstration Center for Experimental Plant Science Education, College of Agriculture, Guangxi University, Nanning, China

**Keywords:** heavy metal, phytoremediation, AMF, transcriptome, hyperaccumulation-regulation network

## Abstract

Arbuscular mycorrhizal fungi (AMF) have been widely shown to significantly promote the growth and recovery of *Pteris vittata* L. growth and repair under arsenic stress; however, little is known about the molecular mechanisms by which AMF mediate the efficient uptake of arsenic in this species. To understand how AMF mediate *P. vittata* arsenic metabolism under arsenic stress, we performed *P. vittata* root transcriptome analysis before and after *Claroideglomus etunicatum* (*C. etunicatum*) colonization. The results showed that after *C. etunicatum* colonization, *P. vittata* showed greater arsenic resistance and enrichment, and its dry weight and arsenic accumulation increased by 2.01–3.36 times. This response is attributed to the rapid reduction and upward translocation of arsenic. *C. etunicatum* enhances arsenic uptake by mediating the MIP, PHT, and NRT transporter families, while also increasing arsenic reduction (PvACR2 direct reduction and vesicular PvGSTF1 reduction). In addition, it downregulates the expression of ABC and P-type ATPase protein families, which inhibits the compartmentalization of arsenic in the roots and promotes its translocation to the leaves. This study revealed the mechanism of *C. etunicatum*-mediated arsenic hyperaccumulation in *P. vittata*, providing guidance for understanding the regulatory mechanism of *P. vittata*.

## Highlights

- *Claroidoglomus etunicatum* increased arsenate reductase activity in the roots of *Pteris vittata* and promoted a reduction in arsenic.
- *C. etunicatum* mediated the selective transport of arsenic via transporters.
- *C. etunicatum* endowed *P. vittata* with high resistance to arsenic

## 1 Introduction

Heavy metal pollution in farmland soil has become increasingly serious due to the continuous rise in human economic activities, with arsenic pollution in farmland soil becoming a problem that cannot be ignored. According to the 2014 National Soil Pollution Survey Communique by the Ministry of Environmental Protection and the Ministry of Land and Resources of China, the total excess pollutants in China’s soil (based on China’s secondary Standard for Soil Environmental Quality, GB15618-1995) is 16.1%, with 2.7% representing arsenic pollution (MEP of China (Ministry of Environmental Protection of China) National soil pollution survey bulletin). Notably, the Earth’s crust naturally contains a small amount of arsenic (approximately 2 to 5 mg/kg), and natural factors such as volcanic eruptions and geological movements contribute to the redistribution and migration of arsenic in the soil (Khalid et al., 2017). Moreover, human economic activities lead to large amounts of arsenic entering the soil, intensifying arsenic deposition and adhesion in the soil. Approximately 50,000 tons of arsenic are released annually via human economic activities (Bolan et al., 2014). These activities, along with the use of groundwater with high arsenic concentrations for agricultural irrigation, have intensified the accumulation and hyperaccumulation of arsenic in farmlands (Rahman et al., 2014; Hadzi et al., 2018). Arsenic in soil is easily absorbed by plants and accumulates in the food chain, posing significant risk to human health. Therefore, the restoration of arsenic-contaminated farmland soil is imperative for the sustainable development of agriculture.

Conventional physical and chemical remediation technologies are unsuitable for remediating arsenic pollution in farmlands due to the high treatment costs, negative impacts on soil fertility, and the risk of secondary pollution (Li C. et al., 2019; Rajendran et al., 2022). As a result, phytoremediation technology is considered the most optimal approach for mitigating or eliminating the toxic effects of arsenic contamination in agricultural soils (Bhat et al., 2022). In contrast to physical and chemical restoration methods, phytoremediation offers advantages such as cost-effectiveness and simplicity. However, its efficiency is influenced by factors like the plant growth cycle and genotype (Fasani et al., 2018). In soils with elevated levels of arsenic contamination, limited plant growth hinders phytoremediation effectiveness. Therefore, mitigating the toxic effects of arsenic stress on plants while promoting their growth and enhancing arsenic uptake and accumulation is crucial for improving the efficiency of soil phytoremediation in addressing arsenic pollution in soils.

*Pteris vittata* L. was the first fern discovered to be an arsenic hyperaccumulator, and ferns hold great potential for soil arsenic remediation due to their unique characteristics (Ma et al., 2001; Tu et al., 2002; Wei and Chen, 2006). Previous studies have clarified the mechanism of arsenic hyperaccumulation in *P. vittata*, where As(V) absorbed by the roots is reduced to As(III) in the rhizosphere and then translocated to the leaves for sequestration (Kumar et al., 2016; Bai et al., 2023). Arsenic uptake by *P. vittata* roots occurs mainly through phosphate transporters and aquaporin proteins. The Pht1 family, mainly *PvPht1;3*, is responsible for arsenate uptake, while the TIP class, *PvTIP4;1*, is responsible for arsenite uptake (He et al., 2016; Zhang et al., 2020). Unlike phosphate transporters, plants have many types of aquaporin proteins. Among these, the localization and function of plasma membrane intrinsic proteins (PIP), tonoplast intrinsic proteins (TIP), and nodularin-like endogenous protein (NIP) have been fully described, with NIPs considered the main aquaporin proteins involved in arsenite uptake in plants (Bienert et al., 2008; Tanaka et al., 2008; Maurel et al., 2015). In addition, *PvPTB1;1/1;2* may mediate the secretion of phytic acid, thereby affecting the absorption and transport of arsenic and phosphorus (Sun et al., 2022). In the roots of *P. vittata*, As(III) is preferentially transported to the aboveground parts of plants via As(V) (Wang et al., 2010; Lei et al., 2012). The reduction of arsenate in *P. vittata* involves a variety of proteins. *PvACR2*, *PvHAC1*, and *PvHAC2* are considered to directly mediate arsenate reduction (Ellis et al., 2006; Li X. et al., 2020). In addition, *PvGAPC1*, *PvOCT4*, and *PvGSTF1* are involved in indirect arsenate reduction. *PvGAPC1* first converts arsenate in the cell into 1-As(V)-3-phosphoglycerate, which is then pumped into specific arsenic metabolic vesicles by *PvOCT4* for the release of As(V), and is finally reduced to As(III) by *PvGSTF1* (Ellis et al., 2006; Cai et al., 2019).

Arbuscular mycorrhizal fungi (AMF) can form symbiotic relationships with most plants, enhancing their ability to cope with adversity. AMF improve plant host access to restricted mineral nutrients through an extensive mycelial network while obtaining carbon (C) sources from the plants in return (Jiang et al., 2017; Duan et al., 2024). Therefore, nutrient supply and abiotic stress are the main factors that determine whether AMF produce a net gain in resources. For example, at low phosphorus levels, plants can induce the colonization of AMF to alleviate the symptoms of phosphorus deficiency, resulting in a significant increase in the colonization rate of AMF. However, under high phosphorus (P) conditions, AMF have little effect on plant growth or nutrient uptake, and plants can limit their colonization (Wang et al., 2017; Wipf et al., 2019). Under abiotic stress, plants increase the colonization of AMF through complex mechanisms. Colonized AMF promote water absorption by stimulating changes in plant hormone levels, facilitating hyphal absorption, and employing other mechanisms (Chitarra et al., 2016; Santander et al., 2021). Under conditions of heavy metal stress, AMF can reduce the accumulation of heavy metals in plants by fixing and discharging them. They play an important role in reducing the toxicity of heavy metals in plants, obtaining nutrients, and improving the performance of plants under heavy metal stress (Riaz et al., 2021; Colombo et al., 2024). AMF colonization can upregulate the expression of antioxidant-related genes in plants, including those encoding zinc transporters, metallothioneins, 90 kD heat shock proteins, and glutathione S-transferases, thereby improving the tolerance of mycorrhizal plants to heavy metals (Bona et al., 2011). Under arsenic stress, *P. vittata* exhibits a relatively strong arsenic defense mechanism, resulting in significant increases in both biomass and arsenic accumulation (Yizhu et al., 2020). For example, by upregulating the expression of AMF-induced phosphate transporters (PHTs), the absorption of phosphorus and arsenic by the roots can be increased (Sun et al., 2022).

To date, phytoremediation techniques using *P. vittata* have been employed in remediation projects in certain regions of China (Chen et al., 2018). However, due to the slow growth of the plant and the reliance on single-plant extraction for arsenic contamination remediation, the remediation cycle is lengthy, making it difficult to promote large-scale applications (Matzen et al., 2022). Therefore, enhancing the phytoremediation efficiency of hyperaccumulators is a prerequisite for promoting the widespread application of *P. vittata* in the remediation of arsenic contamination. The use of AMF to increase crop stress resistance and reduce heavy metal accumulation has been extensively studied (Li Y. et al., 2019; Kaur et al., 2023; Lv et al., 2023). However, relatively few studies have investigated the use of AMF to increase the phytoremediation efficiency of *P. vittata* in arsenic-contaminated soil. Although our previous studies reported that AMF promote the accumulation of arsenic by *P. vittata* through rhizosphere regulation, the physiological and molecular mechanisms have not yet been elucidated (Pan et al., 2022).

In this study, pot experiments were conducted to investigate the effects of *C. etunicatum* colonization on the uptake and transport of arsenic in *P. vittata*, which were grown in arsenic-contaminated soil. Through comprehensive physiological and transcriptome analyses, this study revealed the effects of *C. etunicatum* colonization on the arsenic metabolism and tolerance mechanisms of *P. vittata* under arsenic stress. The results elucidate the physiological and molecular mechanisms by which *C. etunicatum* inoculation promotes arsenic hyperaccumulation in *P. vittata*, providing strategies for more effective arsenic accumulation in phytoremediation applications.

## 2 Materials and methods

### 2.1 Experimental materials

The tested AMF was *Claroideoglomus etunicatum*, which was screened by our research group in the early stage. To culture *C. etunicatum*, sterilized fine sand (substrate) and clover (host) were cultured for 3~4 months. The sand size was ≤ 2 mm, and the clover roots were collected and used as inoculants. The *P. vittata* spores used in the experiment were collected from Hunan, China. Before the experiment, the spores were sown on a sterilized culture medium (organic soil). After germination, the plants were watered once a week with Hoagland nutrient solution. Once the plants had developed four or five leaves, they were used as experimental seedlings. The tested soil was low-phosphorus soil collected from the suburbs of Nanning City, China (the physical and chemical properties are shown in **Supplementary Table S1**). The soil was screened and sterilized (121°C, 30 min), followed by aging with 400 mg kg^−1^ sodium arsenate for 2 months. Before cultivation, nitrogen and potassium fertilizers (N, 200 mg kg^−1^ and K_2_O, 180 mg kg^−1^) were added.

### 2.2 Pot experiment

The plants were divided into two treatment groups, inoculated (CE) and noninoculated (CK), with each treatment replicated three times. At the time of transplanting, fungal sand was applied in three layers, with 20 g of fungal sand in each layer, after which the *P. vittata* seedlings were transplanted into the pots. In the noninoculation treatment, sterilized sand was added in the same manner. The culture period lasted for 150 days, and plant height was measured the day before sampling. The total biomass of the plant samples was measured after they were dried to a constant weight in an oven at 65°C following destructive sampling at 105°C. Rhizosphere soil samples were collected for air drying and sieving (2 mm) to determine the basic physical and chemical properties, arsenic concentration, and soil acid phosphatase activity. Some fresh plant samples were stored at − 80°C for transcriptome analysis and As(III) and As(V) analysis. The remaining roots were soaked in a pH 6.0 phosphate buffer at 4°C for 20 min to remove arsenic adsorbed on the root surface.

### 2.3 Sample analysis

#### 2.3.1 Mycorrhizal colonization rate and element content in plants

A small amount of fresh sample was collected and washed with 2% KOH (w/v) in a 95°C water bath for 30 min, followed by acidification in diluted hydrochloric acid for 30 min. In accordance with the methods of Li et al (Jansa et al., 2008), the samples were stained with 0.05% trypan blue solution, and the colonization rate of mycorrhizae was determined under a microscope. After drying and crushing, the plant tissue samples were subjected to microwave digestion with concentrated HNO_3_ (GR, Mars 6, CEM Company, USA), and the arsenic content was analyzed via inductively coupled plasma spectrometry (ICP-5000, Spotlight Technology Co. Ltd., Hangzhou, China) The recovery rate of arsenic in the analysis process was 97%. The plant tissue samples were digested with concentrated H_2_SO_4_ (GR), the nitrogen content was determined by the semi-micro Kjeldahl method, and the phosphorus and potassium contents were determined using inductively coupled plasma spectrometry.

#### 2.3.2 Analysis of arsenic(III) and arsenic(V) in plants

The methods of Camurati et al. (2021) were used to analyze the As(III) and As(V) contents in *P. vittata* roots and shoots. Specifically, a *P. vittata* tissue sample, which had been stored at − 80°C after sampling, was removed and freeze-dried (Alpha-4 LD plus, Christ Company, Germany). After freeze-drying, the plant samples were ground and crushed, with liquid nitrogen continuously added during the grinding process. After crushing, 50 mg (accurate to 0.1 mg) of the sample was weighed into a 50-mL centrifuge tube. Next, 20 mL of 0.15 mol L^−1^ HNO_3_ solution was added, and the sample was allowed to stand overnight at room temperature. Afterward, the mixture was heated for 2.5 h in a 90°C incubator, shaking for 1 min every 30 min. After extraction, the mixture was centrifuged at 8,000 r min^−1^ for 15 min, after which the supernatant was removed. The concentration of the 0.45-μM water system filter membrane was tested using liquid chromatography inductively coupled plasma–mass spectrometry (LC-ICP-MS). The recovery rate of arsenic in the analysis process was 92%.

#### 2.3.3 Analysis of the arsenic fractions in *P. vittata* plants

The arsenic in the *P. vittata* samples was fractionated using different extractants and divided into three forms: ethanol extract, hydrochloric acid extract, and residue. The arsenic content was analyzed using a two-step continuous extraction method. For ethanol extraction, 0.2 g of the plant sample was weighed, 10 mL of 80% ethanol solution was added, and the mixture was shaken at 25°C for 20 h. Subsequently, the mixture was centrifuged at 8,000 r min^−1^ for 15 min. The liquid was removed, and 10 mL of 80% ethanol solution was added again. The mixture was shaken at 25°C for 2 h. The mixture was centrifuged twice, and the liquid was collected three times. The temperature gradient was then increased for heating (80°C for 30 min and 100°C for 30 min). Next, 2 mL of concentrated HNO_3_ solution was added, and the mixture was covered and refluxed for 2 h. The acid was removed, and the volume was fixed for analysis. For hydrochloric acid extraction, 10 mL of 0.6 mol L^−1^ hydrochloric acid solution was added to the residue in the centrifuge tube after extraction with 80% ethanol. The mixture was shaken for extraction at a constant concentration and volume, following the methods described above. The residue was dried at 65°C until a constant weight was achieved, and its weight was analyzed according to the method described in **Section 2.3.1**. The recovery rate of arsenic during the analysis process was 88%–92%.

#### 2.3.4 Analysis of arsenate reductase activity in *P. vittata* roots

One gram of fresh *P. vittata* root was weighed, 10 mL each of 50 mM 3-(*N*-morphorphine) propanesulfonic acid and 50 mM MES buffer solution (pH 6.5) were added, and a small amount of quartz sand was added. The mixture was ground to a homogenate at 4°C, centrifuged at 4°C for 30 min (10,000 r min^−1^), filtered, and passed through a Sephadex PD-10 chromatography column. A plant arsenate reductase (AR) kit (Shanghai Shuhua Biological Company) was used for the analysis according to the manufacturer’s instructions.

#### 2.3.5 X-ray photoelectron spectroscopy

The *P. vittata* tissue samples stored at − 80°C were freeze-dried, crushed, sieved through 200 mesh, and pressed into tablets. The samples were analyzed using photoelectron spectroscopy (K-Alpha, Thermo Scientific, USA). The electron binding energy was corrected by the carbon oxide C 1s peak (284.8 eV).

#### 2.3.6 RNA extraction, library preparation, sequencing, and read mapping

Total RNA was extracted from *P. vittata* root tips using TRIzol reagent (Invitrogen Co., Carlsbad, CA, USA) according to the manufacturer’s instructions. The RNA mass was analyzed using a 5300 bioanalyzer (Agilent Co., Santa Clara, CA, USA) and quantified using an ND-2000 (Thermo Fisher NanoDrop, Waltham, MA, USA). High-quality RNA samples (OD260/280 = 1.8–2.2, OD260/230 ≥ 2.0, RIN ≥ 6.5, 28S:18S ≥ 1.0, > 1 μg) were used to construct sequencing libraries, which were subsequently verified by quantitative real-time PCR (qRT-PCR). RNA purification, reverse transcription, library construction, and sequencing were completed by Shanghai Maggio Biomedical Biotechnology Co. Ltd. (Shanghai, China).

The raw paired-end reads were trimmed and quality controlled via fastp with default parameters. The clean data from the samples were subsequently used for *de novo* assembly with Trinity. To increase the assembly quality, all the assembled sequences were filtered with CD-HIT and TransRate and assessed with Benchmarking Universal Single-Copy Orthologs (BUSCO). The assembled transcripts were searched against the National Center for Biotechnology Information (NCBI) protein nonredundant (NR), Clusters of Orthologous Groups of proteins (COG), and Kyoto Encyclopedia of Genes and Genomes (KEGG) databases using Diamond to identify the proteins that had the highest sequence similarity with the given transcripts to retrieve their functional annotations. Typical cutoff E values were set at less than 1.0 × 10^−5^. The BLAST2GO program was used to generate Gene Ontology (GO) annotations of unique assembled transcripts for describing biological processes, molecular functions, and cellular components.

#### 2.3.7 Differential expression and gene set analysis

To identify differentially expressed genes (DEGs) between different treatments, the expression level of each transcript was calculated according to the fragments per kilobase of the exon model per million mapped fragments method (FPKM). RNA-seq by expectation maximization (RSEM) was used to quantify gene abundance. DESeq2 was used for differential expression analysis. DEG genes with a |log2FC|≥1 and a false discovery rate (FDR) < 0.05 were considered significantly differentially expressed genes. In addition, functional hyperaccumulation analysis, including GO and KEGG analyses, was performed to identify which DEGs were significantly enriched in GO terms and metabolic pathways at a Bonferroni-corrected *p*-value < 0.05 compared with the whole-transcriptome background. GO functional hyperaccumulation and KEGG pathway analyses were carried out using Goatools and Python SciPy software, respectively.

In accordance with the methods of Yan et al (Yan et al., 2019), the keyword “transport” was used to screen transporters from DEGs and identify the main transporter families related to arsenic metabolism. The genes involved in protein degradation were identified from KEGG pathways map04141, map04120, and map03050. The enzymes involved in ROS and glutathione (GSH) metabolism were screened through KEGG pathways map00053 and map00480, respectively. When conducting gene set enrichment analysis (GSEA), to map as many genes as possible, all genes annotated in the KEGG analysis were used as a prior gene set when analyzing protein degradation, ROS, and GSH metabolism.

#### 2.3.8 Quantitative real-time PCR validation

Using identical RNA/cDNAs for RNA-seq as templates, qRT-PCR was performed on a Bio-Rad CFX96 instrument (Bio-Rad Laboratories, Hercules, CA, USA) to verify the authenticity of the transcriptomic profile expression patterns. Eleven candidate DEGs involved in different processes were selected as target genes, and the housekeeping gene *PvActin* was used as an internal control. The primers used for qRT-PCR are listed in **Supplementary Table S2**. The fluorescent qPCR system included 16.5 μL of 2 × SYBR Select Master Mix (Thermo Fisher Scientific), 0.8 μL each of forward and reverse primers, 2 μL of cDNA template, and water added to bring the final volume to 20 μL. The instrument used was an ABI 7500 fluorescent qPCR instrument (Applied Biosystems, USA), and the reaction proceeded as follows: an initial step at 95°C for 5 min, denaturation at 95°C for 5 s, annealing at 55°C for 30 s, and extension at 72°C for 40 s. The relative expression was calculated using the 2^−ΔΔCT^ formula.

### 2.4 Phytoremediation efficiency evaluation

The extraction amount (EXT), bioaccumulation factor (BAF), translocation factor (TF), and effective translocation factor (ETF) of arsenic were calculated as follows (Zhang et al., 2023):


$$\text{EXT}\left(\text{mg}\right)=\frac{\text{As concentration in plant}\left({\text{mg kg}}^{-1}\right)\times \text{biomass}\left(\text{g}\right)}{1000}$$

$$\text{BAF}=\frac{\text{As concentration in shoot}\left({\text{mg kg}}^{-1}\right)}{\text{As concentration in soil}\left({\text{mg kg}}^{-1}\right)}$$

$$\text{TF}=\frac{\text{As concentration in shoot}\left({\text{mg kg}}^{-1}\right)}{\text{As concentration in root}\left({\text{mg kg}}^{-1}\right)}$$

$$\text{ETF}=\frac{\text{As accumulation in shoot}\left({\text{mg kg}}^{-1}\right)}{\text{As accumulation in root}\left({\text{mg kg}}^{-1}\right)}$$

### 2.5 Statistical analysis

The experimental data were statistically analyzed using Microsoft Excel 2010 and SPSS 21.0, and a *t*-test was used to compare any two samples to test the significance of differences between treatments (*p* < 0.05). The X-ray photoelectron spectroscopy (XPS) experimental data were fitted using Avantage software for peak fitting. All charts were drawn using Origin 2018.

## 3 Results

### 3.1 Effects of *C. etunicatum* on the biomass and nutrient content of *P. vittata*

**Figure 1:**
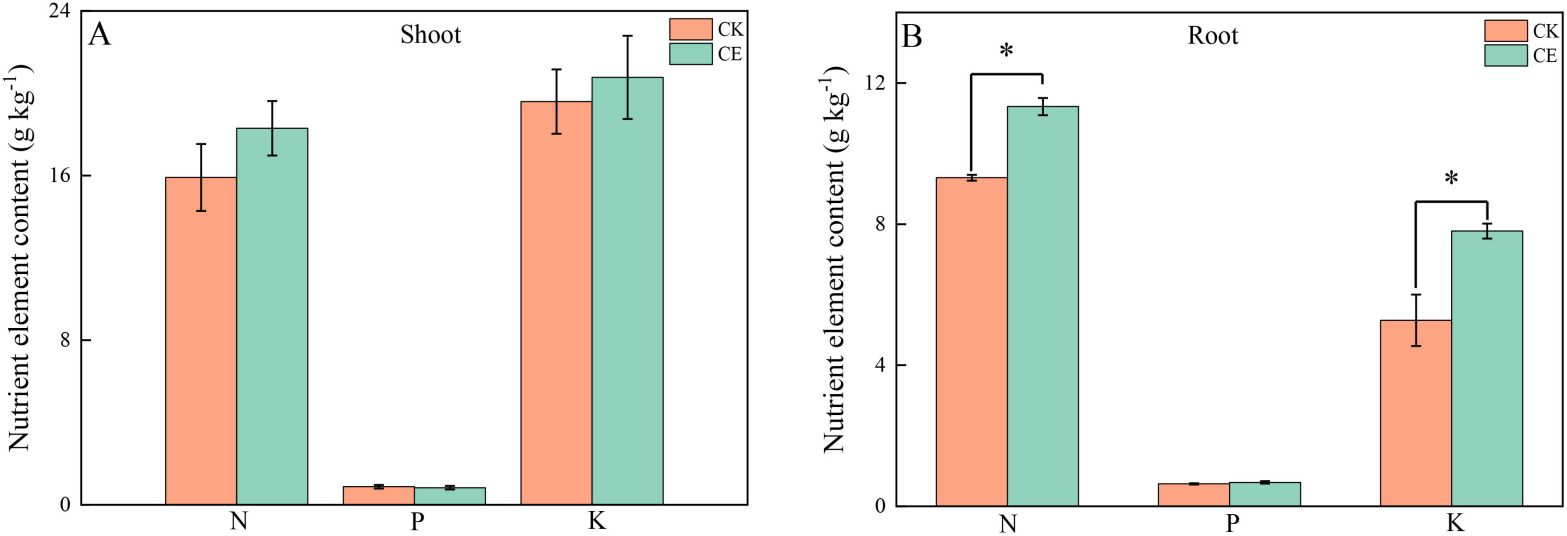
N/P/K contents in different parts of P. vittata under different treatments. **(A)** Shoot; **(B)**-Root. *p < 0.05—significant difference between treatments.

As shown in **Table 1**, under arsenic stress, *C. etunicatum* colonization significantly increased *P. vittata* biomass. Compared with that of the uninoculated treatment (CK), *C. etunicatum* colonization significantly increased *P. vittata* plant height by 1.27-fold, respective fresh weights of the shoots and roots by 2.15- and 1.77-fold, and dry weights by 2.34- and 2.01-fold. Moreover, *C. etunicatum* colonization increased *P. vittata’s* acquisition of elemental nutrients (**Figure 1**). After colonization with *C. etunicatum*, the contents of nitrogen and potassium in the roots significantly increased by 1.22- and 1.48-fold, respectively. The contents of nitrogen and potassium in the shoots slightly increased but did not reach a significant level.

**Table 1:** Effects of different treatments on the biomass of *P. vittata*.

Treatment	Height (cm)	Fresh weight (g)		Dry weight (g)	
		Shoot	Root	Shoot	Root
CK	39.95 ± 0.67 b	8.83 ± 0.11 b	11.56 ± 0.32 b	2.33 ± 0.12 b	1.88 ± 0.31 b
CE	50.90 ± 1.02 a	19.02 ± .80 a	20.42 ± 0.16 a	5.45 ± 0.29 a	3.78 ± 0.36 a

Different letters in the same column indicate significant differences (*p* < 0.05).

### 3.2 Effects of *C. etunicatum* on arsenic accumulation in *P. vittata*

**Figure 2:**
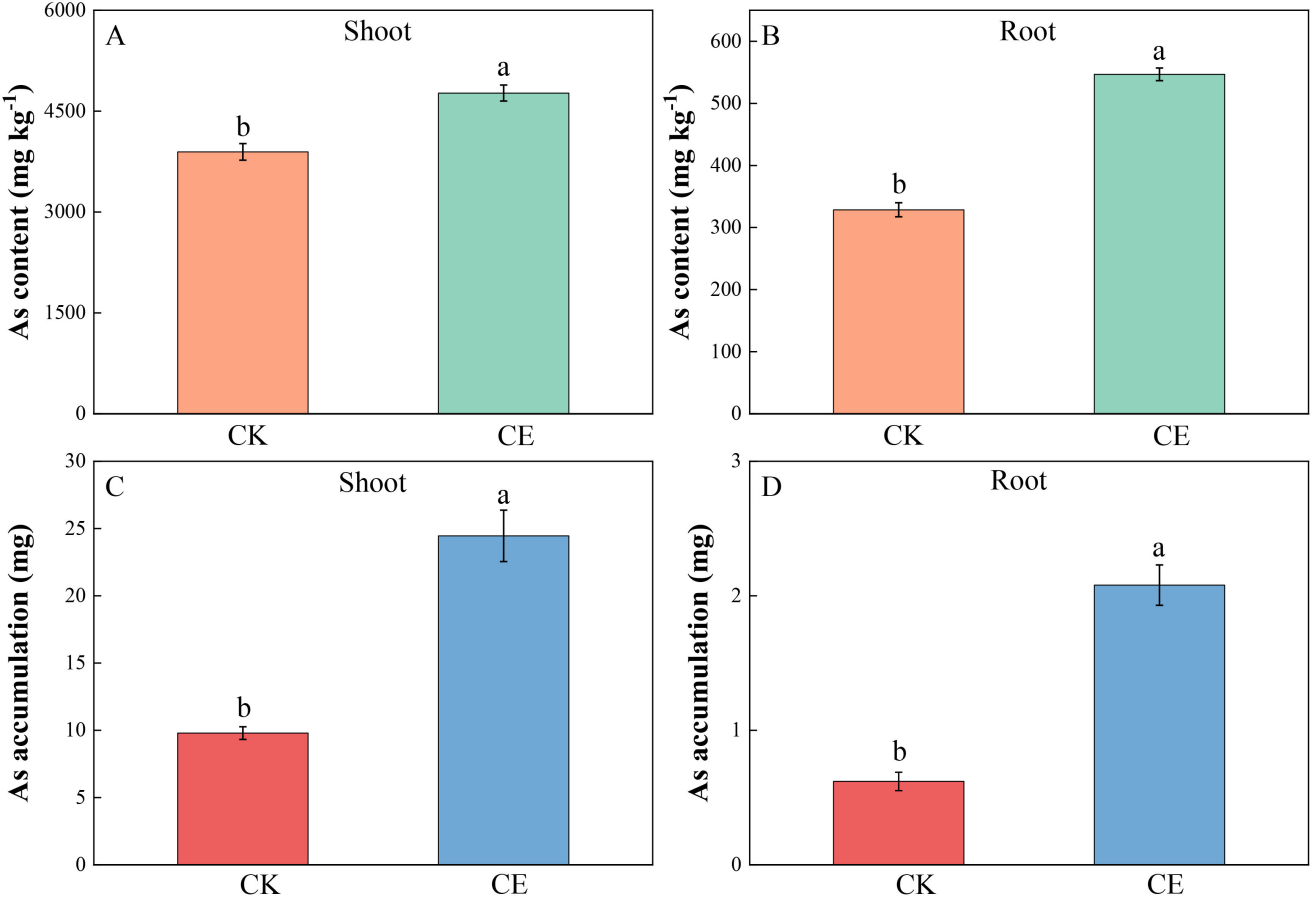
Arsenic content in different parts of P. vittata under different treatments. **(A)** Shoot As content; **(B)** Root As content; **(C)** Shoot As accumulation; **(D)** Root As accumulation. Different letters indicate differences in processing (p < 0.05).

As shown in **Figure 2**, under arsenic stress, *C. etunicatum* colonization significantly increased the arsenic content in the aerial parts and roots of *P. vittata*. Compared with those in the CK, the respective arsenic concentrations in the *P. vittata* aerial parts and roots after *C. etunicatum* colonization were 4,768.36 mg kg^−1^ and 546.73 mg kg^−1^ and were significantly greater by 1.22- and 1.66-fold. *C. etunicatum* colonization also significantly increased the accumulation of arsenic in the shoots and roots of *P. vittata*. After inoculation with *C. etunicatum*, the respective accumulation of arsenic in the shoots and roots of *P. vittata* was 24.46 mg and 2.08 mg, which represented significant increases of 2.69- and 3.36-fold.

**Figure 3:**
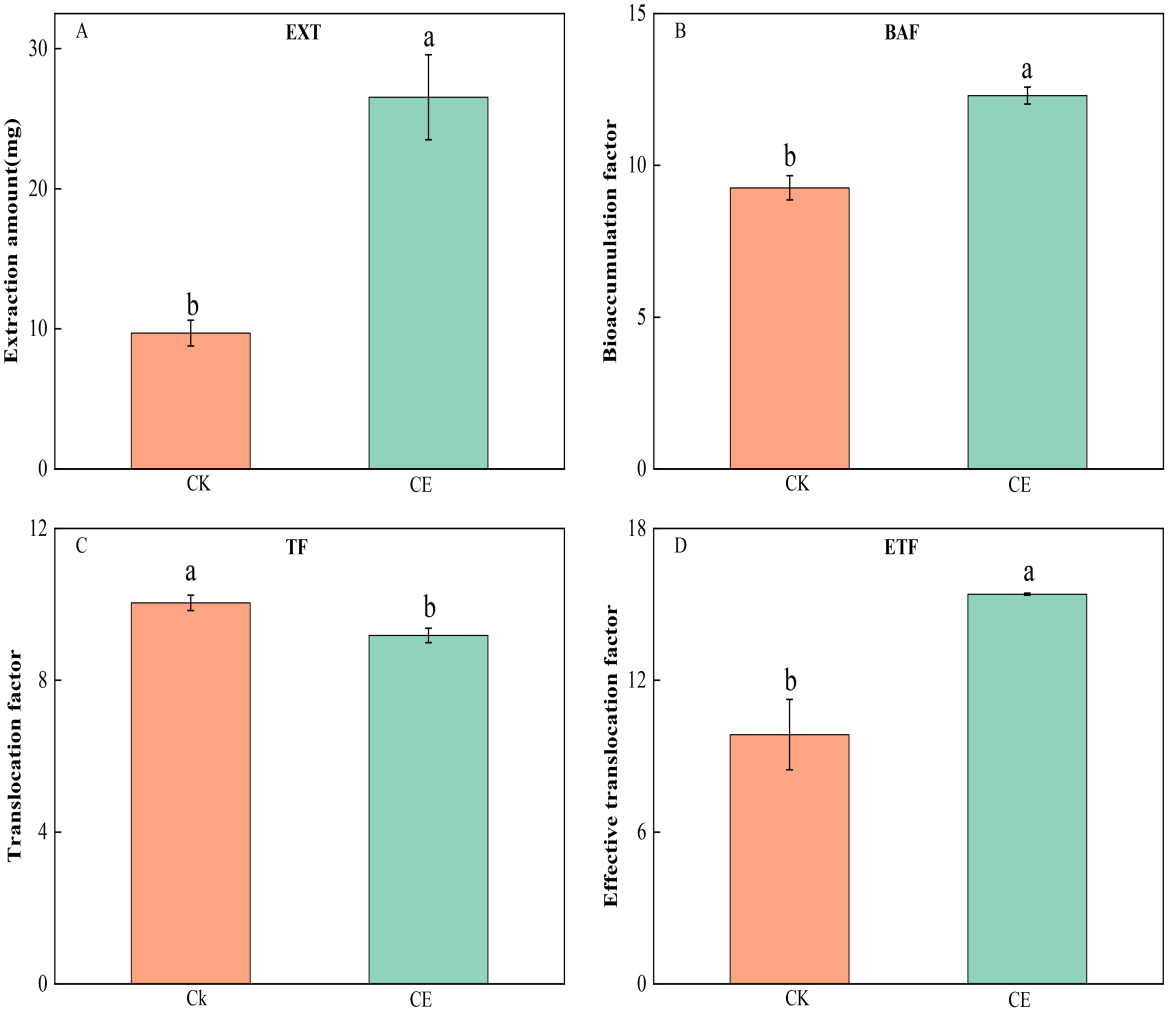
Effect of *C. etunicatum* on the extraction amount **(A)**, bioconcentration factor **(B)**, transformation factor **(C)**, and effective transformation factor **(D)** of *P. vittata*. Different letters indicate differences in processing (p < 0.05).

**Figure 3** shows that *C. etunicatum* colonization greatly improved the remediation efficiency of *P. vittata* on arsenic-contaminated soil. The *P. vittata* arsenic EXT after *C. etunicatum* colonization increased from 9.70 to 26.53, which was 2.74 times greater than that without *C. etunicatum* colonization. The BAF increased by 1.33-fold from 9.26 to 12.30. The effective ETF increased from 9.85 to 15.4, an increase of 1.56-fold. Notably, *C. etunicatum* colonization slightly reduced the arsenic TF in *P. vittata*.

### 3.3 Effects of *C. etunicatum* on arsenic speciation in *P. vittata*

**Figure 4:**
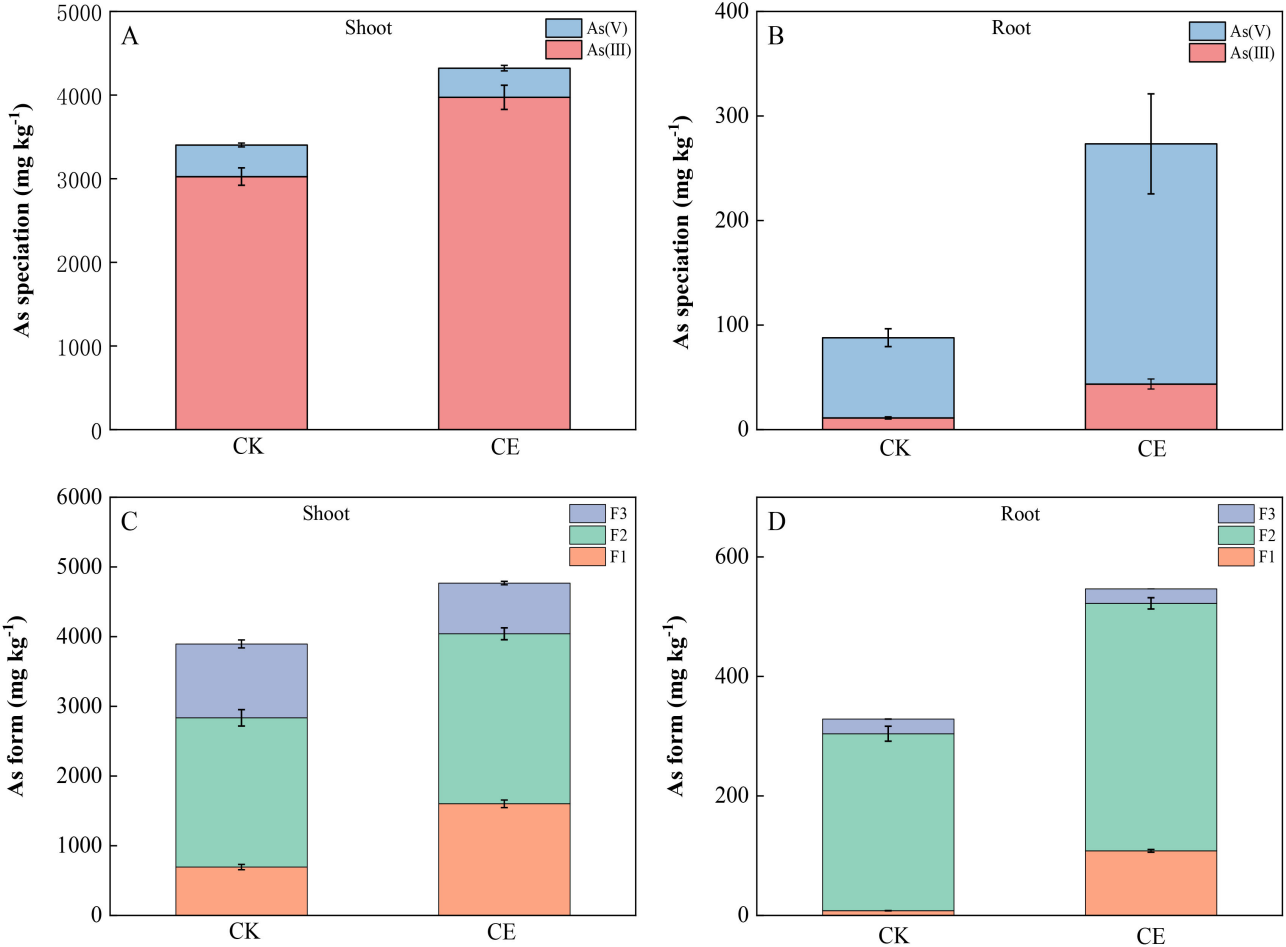
Effect of *C. etunicatum* colonization on arsenic speciation in different parts of *P. vittata.* **(A, B)** Shoot and root arsenic valence states; **(C, D)** Arsenic extracted from shoots and roots. F1, ethanol extraction form; F2, hydrochloric acid extraction form; F3, residual.

As shown in **Figure 4**, the *P. vittata* stems mainly contained As(III), whereas the roots mainly contained As(V). The colonization of *C. etunicatum* significantly increased the content of As(III) in *P. vittata* shoots and roots. After *C. etunicatum* colonization, the shoot As(III)/As(V) ratio increased from 7.92 to 11.14, a significant increase of 1.41-fold, and the root As(III)/As(V) ratio increased from 0.16 to 0.20. Different extractants were used to fractionate the *P. vittata* shoots and roots (**Figures 4C, D**. The *P. vittata* shoots and roots contained mainly F2 arsenic (extracted with hydrochloric acid), accounting for more than 51% of arsenic, and almost all the roots contained F2 arsenic, accounting for 76%~97% of the total. *C. etunicatum* colonization significantly changed the proportion of arsenic in the three different forms of *P. vittata*. Compared with those in the CK treatment, the proportion of F1 arsenic in the shoots of *P. vittata* after *C. etunicatum* colonization increased by 17%, the proportion of F3 arsenic decreased by 12%, and the proportion of F2 arsenic changed minimally. Similarly, *C. etunicatum* colonization significantly increased the proportion of F1 arsenic in *P. vittata* roots by 18% but significantly reduced the proportion of F2 arsenic in the roots (by 17%).

**Figure 5:**
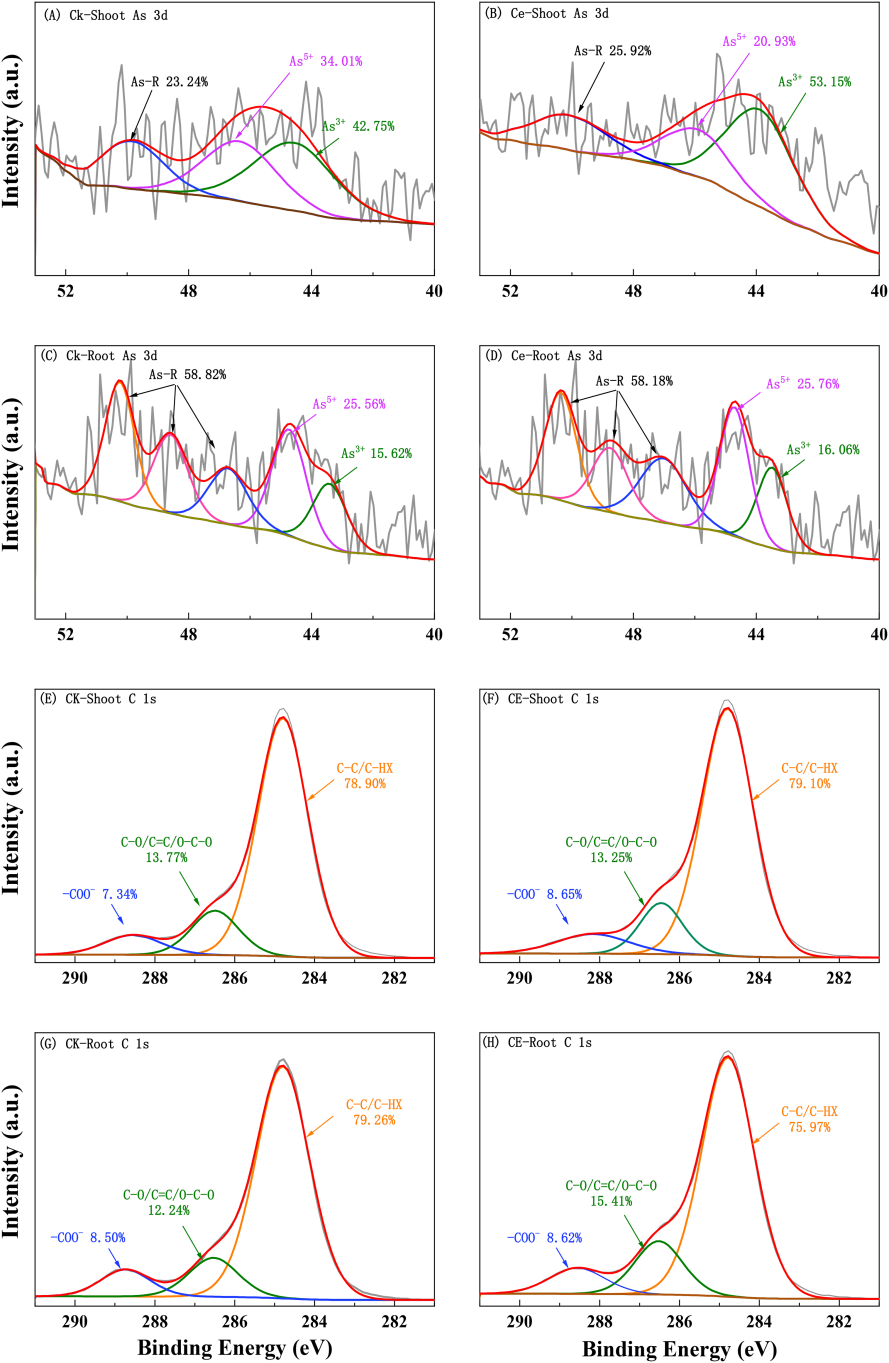
XPS analysis spectra of *P. vittata* under arsenic stress. **(A–D)** Root; **(G–H)** shoot.

The XPS absorption spectrum and peak fitting are shown in **Figure 5**. The arsenic in the roots was mainly As(V) and contained a large amount of high-binding energy arsenic (As-R). This high-binding energy arsenic may be in a complex state with organic matter. After *C. etunicatum* colonization, the relative content of As(III) in *P. vittata* roots increased. In contrast, the *P. vittata* shoots contained mainly As(III), and the proportion of high-binding energy arsenic content was lower than that in the roots. However, *C. etunicatum* colonization also increased the relative content of As(III) in *P. vittata* shoots. Both the *P. vittata* roots and shoots contained three forms of carbon (C–C/C–HX, C–O/C=C/O–C–O, and –COO–). Compared with the relative content of COO in the CK, after *C. etunicatum* colonization, the COO content in the *P. vittata* roots and shoots increased, the content of C–O/C=C/O–C–O in the roots increased, and the content of C–O/C=C/O–C–O in the shoot decreased.

### 3.4 Arsenate reductase activity and sulfur content in roots

**Figure 6:**
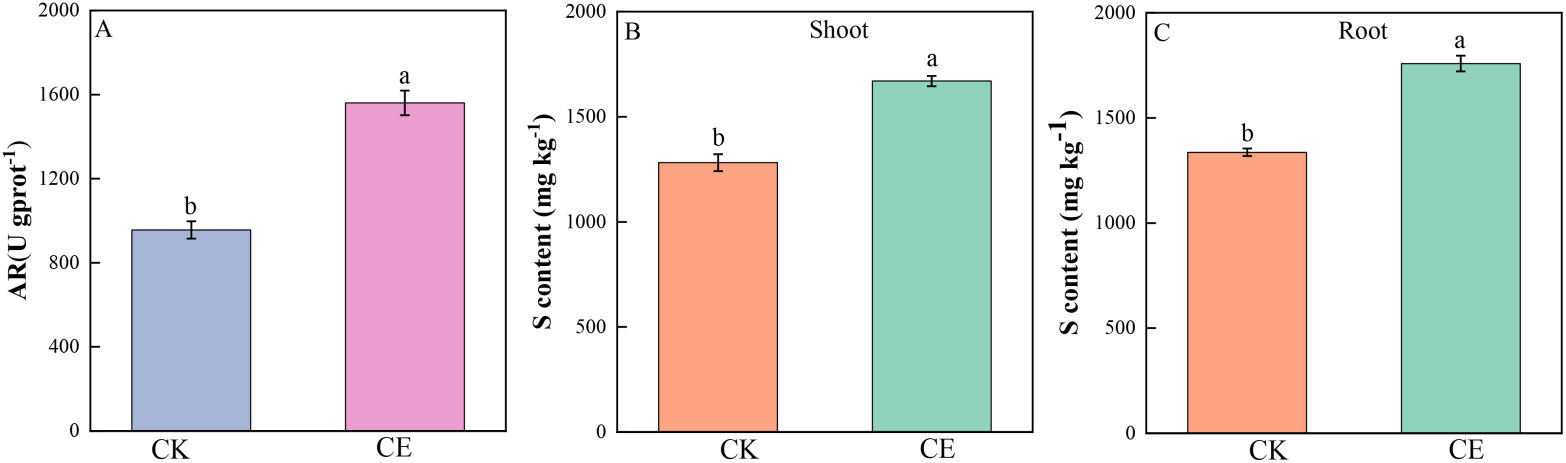
Sulfur content and root AR activity of *P. vittata*. **(A)** Arsenate reductase activity in roots; **(B, C)** Sulfur content in shoots and roots. Different letters indicate differences in processing (p < 0.05).

**Figure 6** shows that *C. etunicatum* colonization significantly increased the arsenate reductase activity of *P. vittata* roots, which significantly increased by 1.85-fold over that of uninoculated roots. Sulfur plays an important role in arsenic metabolism and is directly or indirectly involved in the detoxification mechanism of arsenic in *P. vittata*. The sulfur contents of *P. vittata* shoots and roots significantly increased by 1.36- and 1.30-fold, respectively, after *C. etunicatum* colonization.

### 3.5 RNA-Seq analysis of the *P. vittata* root system under arsenic stress

To reveal the response of the *P. vittata* gene expression profile to *C. etunicatum* colonization under arsenic stress, cDNA library construction and transcriptome analysis was carried out using root samples under arsenic stress that were inoculated or uninoculated with *C. etunicatum*. The Q20 and Q30 values of all clean reads exceeded 97.87% and 93.73%, respectively, the error rate was less than 0.025%, and the mapping rate exceeded 74% (**Supplementary Table S3**), indicating that the sequencing quality met the requirements of the subsequent analysis. The original data from all the transcriptome libraries were stored in the NCBI Sequence Read Archive (SRA) under accession number PRJNA1118441.

#### 3.5.1 *P. vittata* gene profiling under arsenic stress

**Figure 7:**
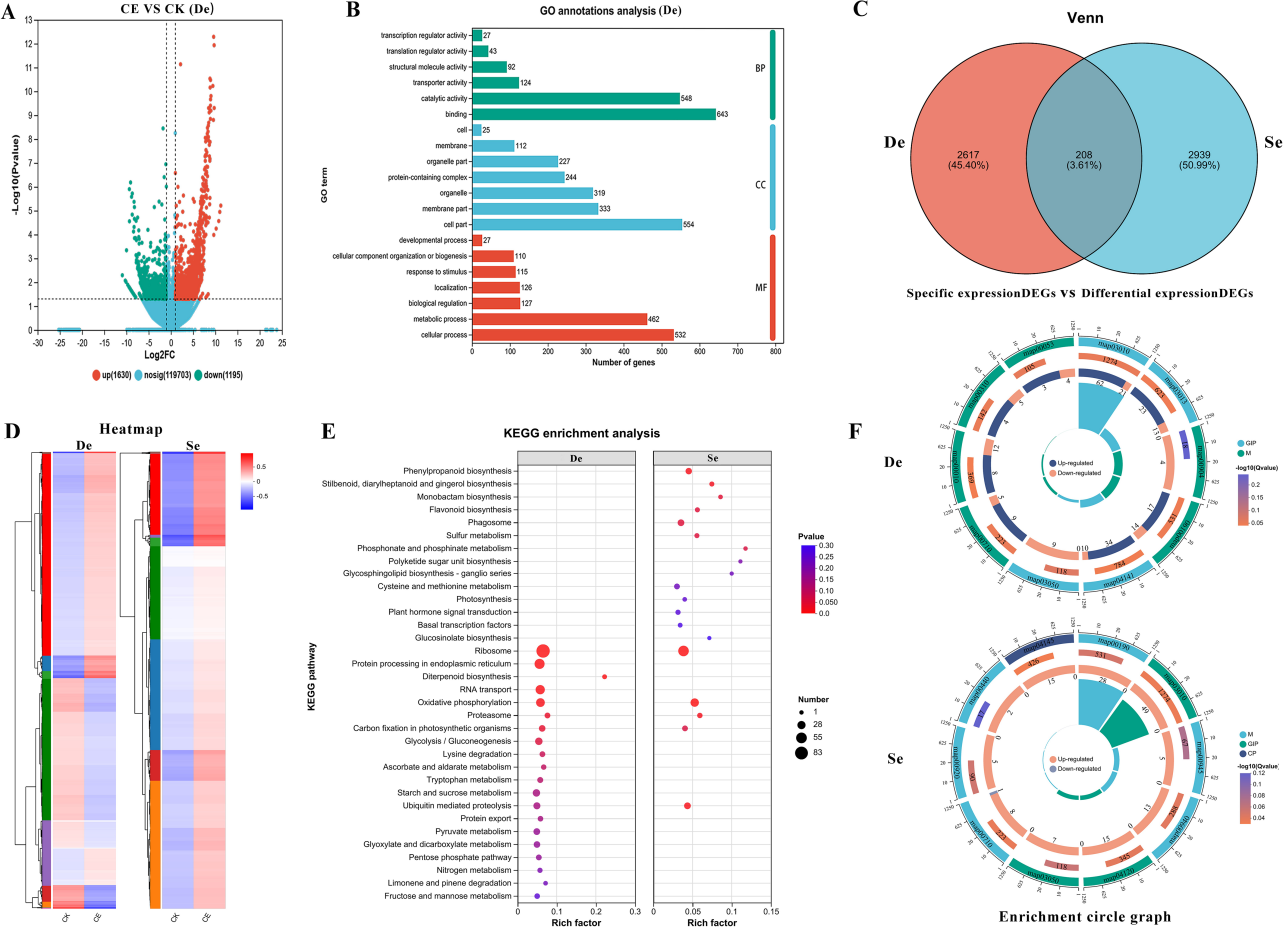
Analysis of differentially expressed genes (DEGs) and specifically expressed genes (DEGs). De, differentially expressed genes (DEGs); Se, CE treatment-specific expressed DEGs. **(A)** Differential gene volcano plot. **(B)** GO annotation results; **(C)** Venn diagram of differentially expressed genes and CE treatment-specific expressed genes. **(D)** Clustered expression heatmap. **(E)** KEGG enrichment analysis. **(F)** Top 10 differential maps by KEGG enrichment degree, comprising four circles from outer to inner: the first circle represents the enriched categories, with the number of genes shown outside the circle on a coordinate scale. Different colors represent different categories. The second circle shows the number of genes in each category in the background and the *p*-value. The more genes, the longer the bar, the smaller the value, and the redder the color. The third circle displays a bar graph showing the proportion of up- and downregulated genes. Green represents the proportion of upregulated genes, and light purple represents the proportion of downregulated genes. The fourth circle shows the enrichment factor for each category (calculated as the number of differentially expressed genes in the category divided by the number of background genes).

To determine the effects of *C. etunicatum* colonization on the expression of related genes in *P. vittata* under arsenic stress, the DEGs were compared and screened (**Supplementary Table S4**). After *C. etunicatum* colonization, a total of 2,825 genes, including 1,630 upregulated genes and 1,195 downregulated genes, were differentially expressed in *P. vittata* roots (**Figure 7**). To clarify the potential biological functions of the DEGs, GO annotation was performed (*p* < 0.05). The respective 46 and 41 GO terms were divided into three categories: biological processes (BP), cellular components (CC), and molecular functions (MF). After colonization, the following GO terms were enriched: catalytic activity and binding in the BP category; cell parts, organelles, and membrane parts in the CC category; and cellular processes and metabolic processes in the MF category. In addition, GO terms that may play a key role in arsenic metabolism, such as transporter activity, detoxification, and antioxidant activity, were found in different rankings (**Figure 7**).

To identify the biological pathways affected by *C. etunicatum* colonization under arsenic stress, DEGs were functionally annotated using the KEGG database, and hyperaccumulation analysis of the KEGG pathways was performed. After *C. etunicatum* colonization, KEGG analysis revealed that among the 480 pathways, 101 were enriched, and eight pathways were significantly enriched (*p* < 0.05). These latter pathways included the ribosome, protein processing in the endoplasmic reticulum, diterpenoid biosynthesis, RNA transport, oxidative phosphorylation, the proteasome, carbon fixation in photosynthetic organisms, and glycolysis/gluconeogenesis. In addition, several important pathways related to substance transport and stress response, such as glutathione metabolism, endocytosis, and sulfur metabolism pathways, were identified. To determine the specific regulatory effect of *C. etunicatum* colonization on *P. vittata* root genes, a Venn diagram was constructed from the samples (the expression levels of DEGs were FPKM > 5 and FDR < 0.01). A total of 3,147 (12.29%) DEGs were specifically expressed after *C. etunicatum* colonization, and only 208 overlapped with the DEGs (**Figure 7D**; **Supplementary Table S5**). The clustering heatmaps of the DEGs and specifically expressed gene sets were significantly different (**Figure 7E**). The KEGG analysis of the specifically expressed DEGs revealed that in addition to having the same KEGG pathway as the differentially expressed DEGs, genes involved in stilbenoid, diarylheptanoid, and gingerol biosynthesis; phenylpropanoid biosynthesis; sulfur metabolism; phosphate and phosphate metabolism and other pathways were also enriched, possibly because *C. etunicatum* mediates arsenic uptake and transport in *P. vittata* roots.

#### 3.5.2 *C. etunicatum*-mediated expression of arsenic transport-related DEGs

**Figure 8:**
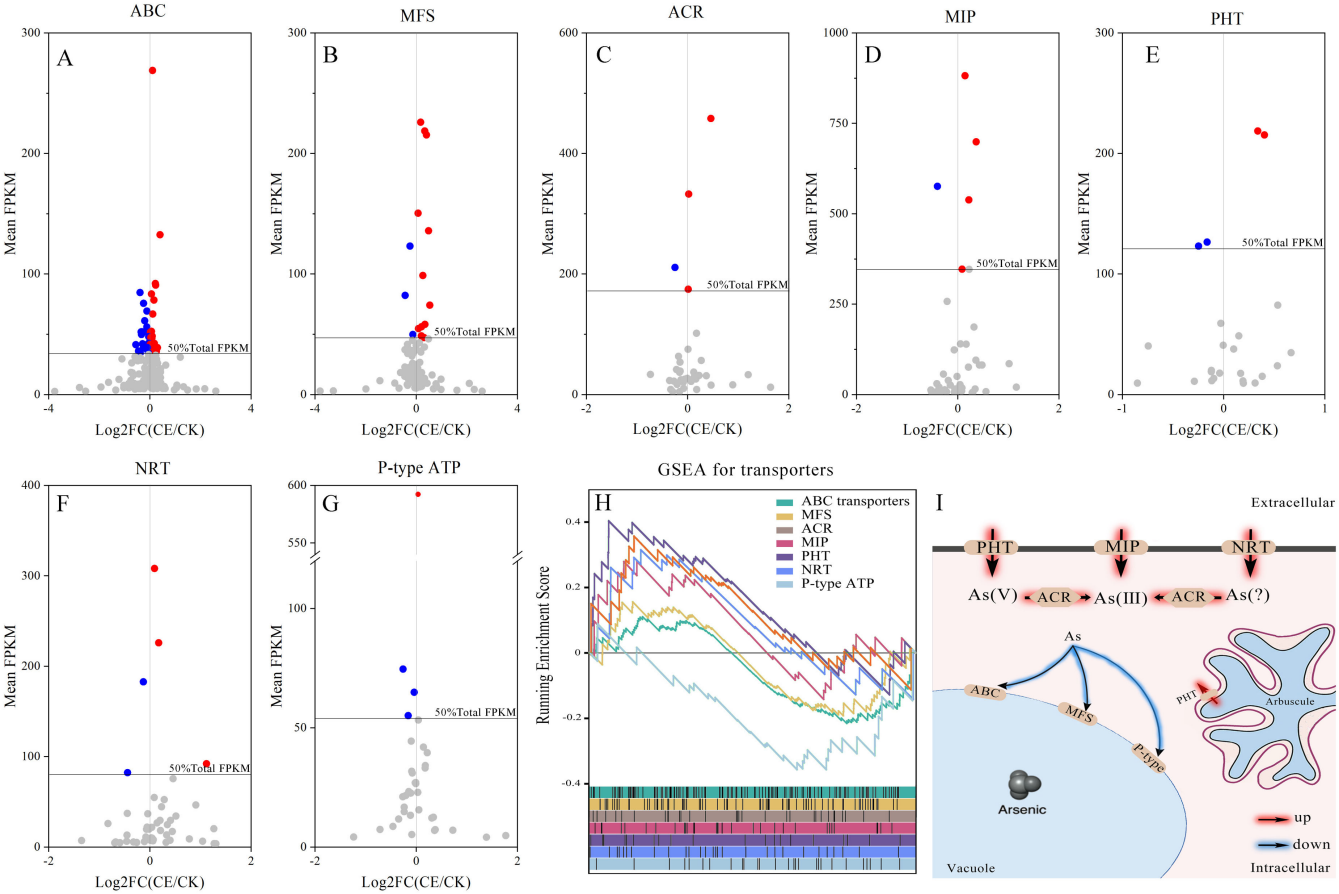
Expression profile of key DEG families involved in arsenic metabolism. **(A–G)** The expression profile of each transporter family, where the color dot represents the top 50% DEGs based on total FPKM for the transporter. **(H)** GSEA of DEGs with FPKM ≥ 5 for each transporter family (the curve represents the ES value, with vertical lines below indicating the corresponding genes). **(I)** AMF-mediated mechanism of arsenic uptake and transport in *P. vittata*.

To identify the major DEGs related to *C. etunicatum* colonization-mediated arsenic hyperaccumulation in *P. vittata*, the ABC superfamily, Major facilitator superfamily (MFS), Arsenical-resistance protein Acr2 or Acr3 (ACR2/3), Major intrinsic protein (MIP), Phosphate transporter protein (PHT), nitrate transporter (NRT), and P-type ATPase (P-ATPase) annotated in all the databases were screened (**Figure 8**; **Supplementary Table S6**). These families are considered the main arsenic transporters of *P. vittata*. To evaluate the effect of *C. etunicatum* colonization on the expression of genes related to arsenic uptake and transport, the DEGs with FPKM > 5 in each family were analyzed using GSEA. After *C. etunicatum* colonization, the DEGs of the MIP, PHP, and NRT gene sets were significantly upregulated, the DEGs of the P-type ATP gene sets were significantly downregulated, and the DEGs of the ABC transporter and MFS gene sets were not significantly changed (**Table 2**). Except for ABC and MFS, the top 10 DEGs based on the FPKM of other families account for over 60% of the total FPKM in the family (**Figures 8A–G**). The leading edge of the GSEA comprises DEGs with obvious changes in expression after colonization by *C. etunicatum*, which may represent genes mediated by *C. etunicatum* (**Supplementary Table S7**). **Table 3** shows the intersection of the leading edge and top DEGs. These genes may be *C. etunicatum* mediated *P. vittata* is a key gene for arsenic uptake and transport in roots.

**Table 2:** Enrichment analysis of the arsenic uptake- and transport-related DEGs.

Gene set name	Size	ES	NES	*p*-value	*p*-adjust	Rank at MAX	Leading edge
ABC transporters	131	− 0.2154	− 0.9267	0.6210	0.6210	3,147	28
MFS	80	− 0.1878	− 0.7355	0.9433	0.9433	3,321	20
ACR	31	0.2623	0.8906	0.6133	0.6133	2,175	6
MIP	36	0.4005	1.4143	0.0532	0.0532	879	7
PHT	21	0.3264	0.9959	0.4487	0.4487	2,350	7
NRT	28	0.3454	1.1432	0.2949	0.2949	2,041	7
P-type ATP	27	− 0.3463	− 1.1007	0.3267	0.3267	4,094	14

The siize represents the total number of genes in the gene set. The ES value is the enrichment score, indicating the degree of hyperaccumulation of gene set members at both ends of the sorting list. The NES value is the standardized hyperaccumulation score. The *p*-value indicates the result of the statistical test performed on the obtained hyperaccumulation score. *p* adjust is the multiple hypothesis test-corrected *p*-value. The rank at MAX refers to the position of the ES value in the sorted list of the gene set. The leading edge is the gene member that contributes the most to the hyperaccumulation score.

**Table 3:** Intersection of leading edge and TOP DEGs.

Gene ID	Description	FC	log2FC	*p*-value
ABC transporters				
TRINITY_DN21608_c0_g3	ABC transporter C family member 2.	0.76	− 0.39	0.52
MFS				
TRINITY_DN5709_c0_g1	Probable inorganic phosphate transporter 1–4	0.84	− 0.25	0.29
TRINITY_DN10546_c0_g1	High-affinity nitrate transporter 2.1	0.74	− 0.44	0.31
TRINITY_DN18724_c0_g1	Sugar transport protein 7	0.91	− 0.15	0.50
ACR				
TRINITY_DN2794_c2_g1	Dual specificity phosphatase Cdc25	1.38	0.46	0.14
TRINITY_DN16620_c0_g1	Arsenical-resistance protein Acr3	1.01	0.01	0.97
MIP				
TRINITY_DN1610_c1_g1	Aquaporin PIP1-1	1.29	0.36	0.05
TRINITY_DN2830_c0_g1	Unknown	1.25	0.32	0.11
TRINITY_DN3277_c0_g1	Probable aquaporin PIP2-1	2.02	1.01	0.01
PHT				
TRINITY_DN688_c0_g1	Low-affinity inorganic phosphate transporter 3	1.32	0.40	0.15
TRINITY_DN11245_c0_g1	Phosphate-repressible phosphate permease pho-4	1.59	0.67	0.34
NRT				
TRINITY_DN158_c0_g1	Protein NRT1/PTR FAMILY 5.1-like	2.17	1.12	0.00
TRINITY_DN14280_c0_g1	NRT1-PTR FAMILY 6-3 protein	1.37	0.46	0.35
TRINITY_DN13756_c0_g1	NRT1-PTR FAMILY 6-3 protein	1.88	0.91	0.28
P-type ATP				
TRINITY_DN10629_c0_g1	Cation-transporting P-type ATPase	0.83	− 0.26	0.12
TRINITY_DN3558_c0_g1	Putative phospholipid-transporting ATPase 9 isoform X1	0.90	− 0.16	0.31
TRINITY_DN5918_c0_g1	Calcium-transporting ATPase 4, plasma membrane-type	0.93	− 0.10	0.67
TRINITY_DN2110_c0_g1	Probable cation-transporting ATPase 13A3	1.10	0.14	0.28

#### 3.5.3 *C. etunicatum-*mediated expression of arsenic reduction- and compartmentalization-related DEGs

**Figure 9:**
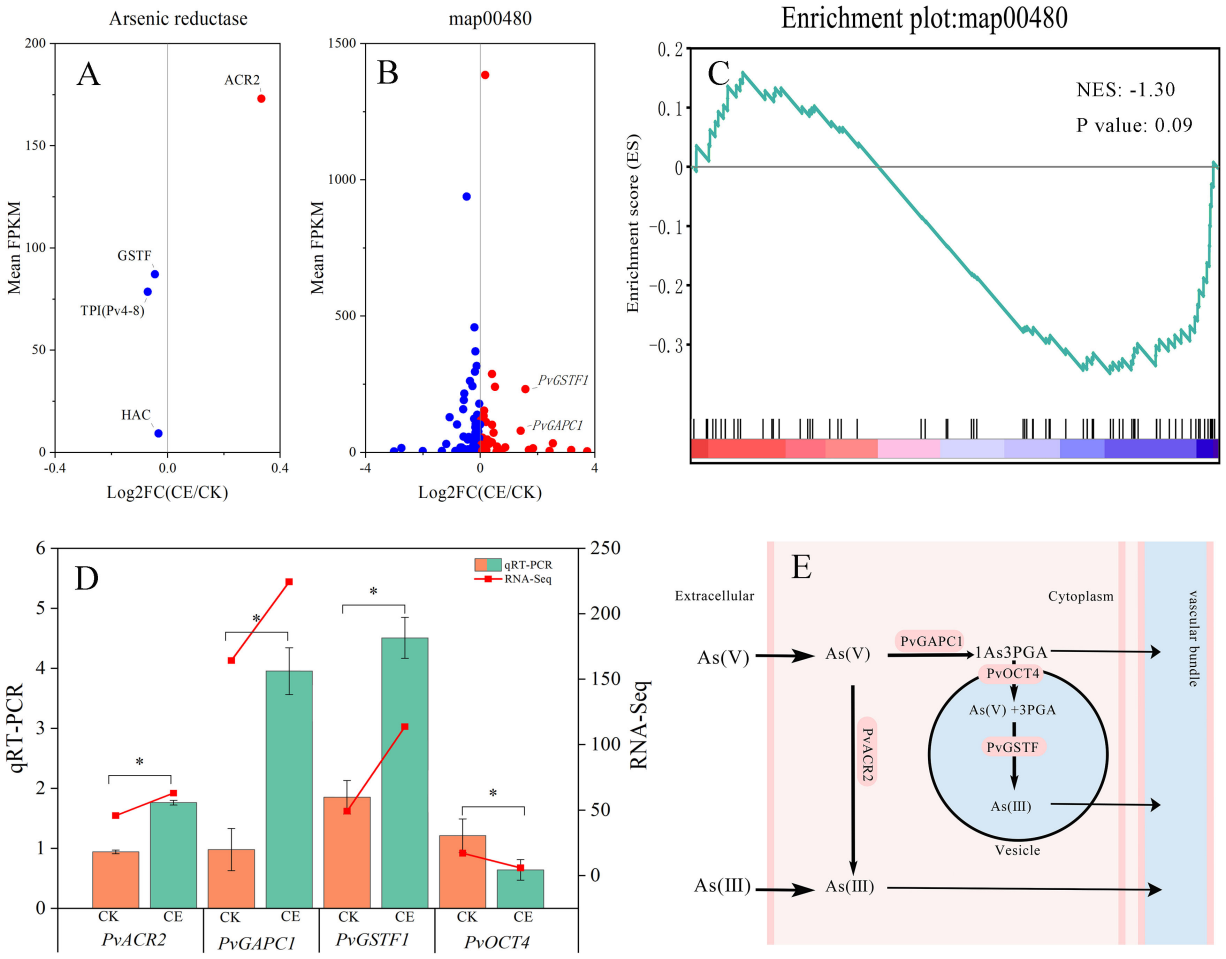
Expression profile of key DEG families involved in arsenic metabolism. **(A)** Transcriptional analysis of arsenic reductase. **(B)** Transcriptional analysis of glutathione metabolic pathway. **(C)** Glutathione metabolic pathway GGSEA. **(D)** Expression of the arsenate reductase gene. **(E)** The pathway of arsenic reduction in roots.

PvACR2, PvHAC1, PvHAC2, Pv4-8, and PvGSTF1 are the arsenate reductases of *P. vittata*. We found that the expression levels of *PvACR2* and *PvGSTF1* were high, and following colonization with *C. etunicatum*, these levels significantly increased. In contrast, the expression levels of *PvHAC1*, *PvHAC2*, and *Pv4-8* were relatively low and did not change significantly (**Figure 9**). The real-time fluorescence quantification results further confirmed that the expression of these arsenic reduction-related genes was significantly upregulated following colonization with *C. etunicatum*. The glutathione metabolic pathway (map00480) plays a role in arsenic reduction and compartmentalization, serving as one of the antiarsenic mechanisms of *P. vittata*. The GSEA results showed that the glutathione metabolic pathway was significantly downregulated following the colonization of *C. etunicatum*, with a total of 25 leading edges (**Supplementary Table S8**). After annotating the leading edge into the KEGG pathway, it was found that the regulated pathway was essentially the same as the DEG pathway (**Supplementary Figure S1**), which included mainly glutathione reduction and NADP+ reduction. Interestingly, although the glutathione metabolic pathway was significantly downregulated, the expression levels of *PvGSTF1* and *PvGAPC1* were significantly upregulated.

#### 3.5.4 *C. etunicatum*-mediated expression of DEGs related to the arsenic resistance pathway

**Figure 10:**
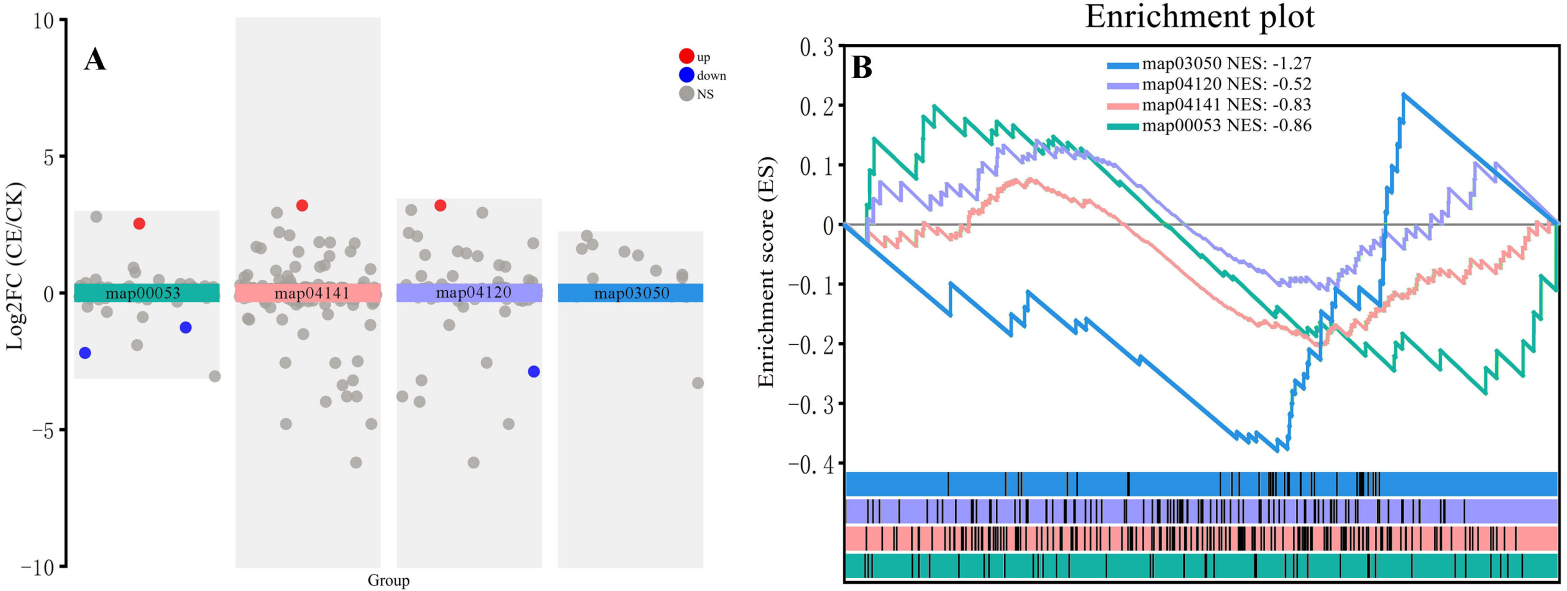
Expression profiles of genes involved in the protein processing in the endoplasmic reticulum (ER)-related degradation (ERAD) pathway. **(A)** Transcriptional analysis of various metabolic pathways. **(B)** Various metabolic pathways GSEA.

Strong ROS metabolism is the main physiological pathway for arsenic resistance. Under arsenic stress, the expression of DEGs related to ascorbic acid and aldehyde metabolism (map00053) was generally high in *P. vittata*, regardless of whether *C. etunicatum* was colonized, and the colonization of *C. etunicatum* did not significantly affect the overall expression of the pathway gene set (**Figure 10**). Protein processing in the endoplasmic reticulum pathway (ERAD, map04141), ubiquitin-mediated proteolysis pathway (UMP, map04120), and proteome pathway (map03050) were used as misfolded process degradation pathways. The GSEA results showed that after *C. etunicatum* colonization, there was no change in the expression of the UMP pathway; however, the ERAD pathway tended to be downregulated, and the proteome pathway was significantly downregulated. These results indicate that the metabolic pressure associated with arsenic resistance in the roots of *C. etunicatum* is relieved after colonization.

#### 3.5.5 Validation of the DEG results by qRT-PCR

**Figure 11:**
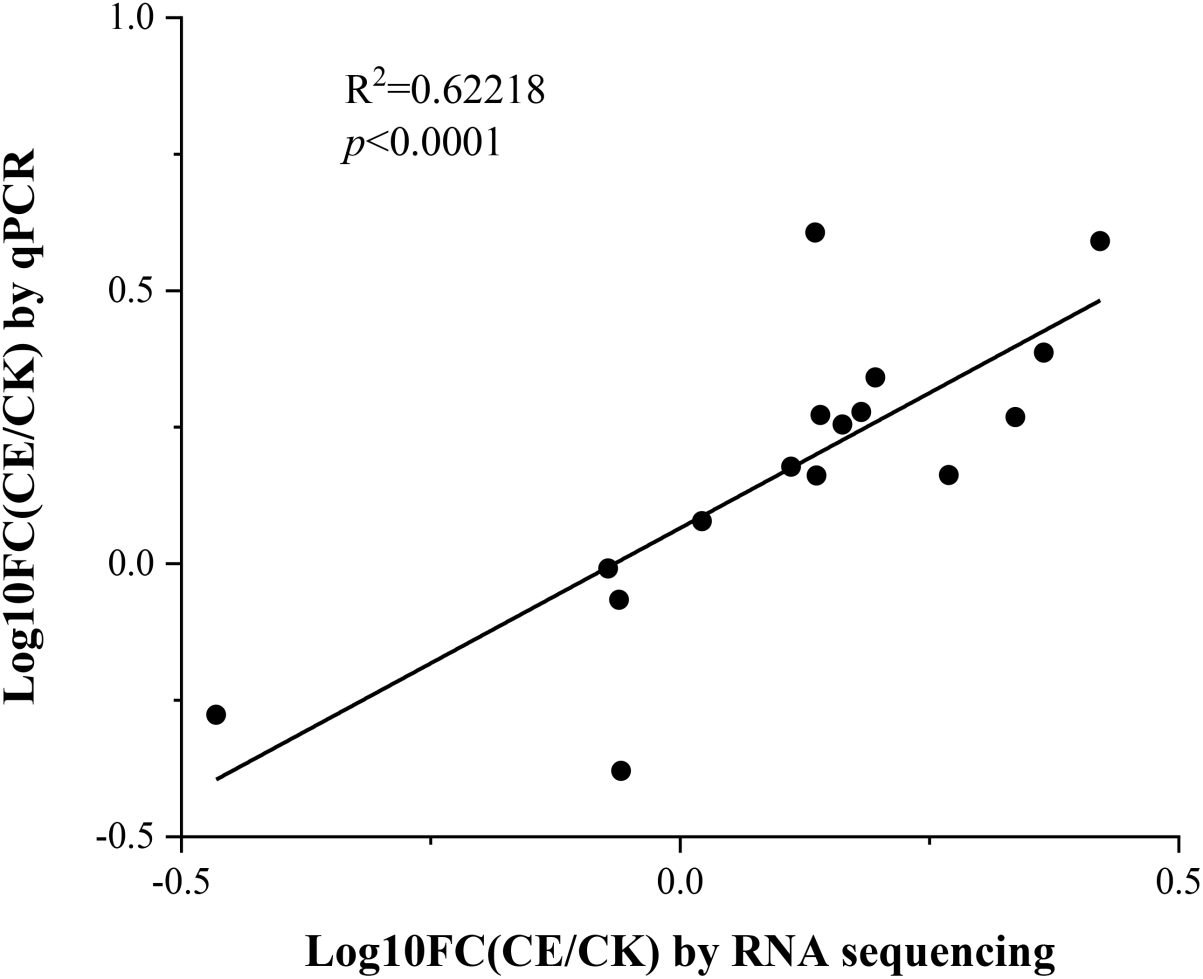
Correlation between qPCR and RNA sequencing for the 16 selected genes. Each point represents the fold change in gene expression level compared to CK.

To confirm the reliability of the transcriptome sequence, we selected 16 key genes involved in various arsenic metabolic pathways and performed real-time quantitative PCR. These genes are mainly associated with arsenic absorption, transport, and detoxification (*PHT1*, *MIP*, *ACR2*, *NRT*, *OCT4*, *GSTF1*, and *GAPC1*). The results showed a strong correlation between the qPCR and RNA sequencing data (*r* = 0.62218, *p* < 0.0001; **Figure 11**). In addition, most of these genes have been clearly identified through genetic transformation. Comparing the expression patterns of these genes before and after colonization by *C. etunicatum* helps explore how this colonization enhances arsenic uptake and hyperaccumulation in *P. vittata*, providing a new perspective.

## 4 Discussion

### 4.1 Effects of *C. etunicatum* colonization on the growth and nutrient acquisition of *P. vittata*

AMF can form symbiotic relationships with most plants, improve host plant nutrition, and increase stress resistance. In this study, under conditions of arsenic stress, the biomass of *P. vittata* significantly increased after inoculation with *C. etunicatum* (**Table 1**), consistent with the findings from previous studies (Leung et al., 2013; Pan et al., 2022). A larger biomass allows for greater accumulation of heavy metals, making the promotion of plant growth a viable strategy to enhance remediation efficiency. The increase in biomass may be partly due to a better nutrient supply. After colonization with *C. etunicatum*, the nitrogen and potassium contents in the roots of *P. vittata* significantly increased, while the nitrogen and potassium contents in the buds showed a slight increase (**Figure 1**). The AMF colonization treatment increased the host P content and had strong effects on the absorption of most other nutrients, particularly Ca, Mg, Na, and S (Horsch et al., 2023). However, due to the shared absorption channels of arsenic and P, as well as the use of low P soil to promote arsenic uptake, no changes in P content were observed (**Figure 1**). Due to the formation of a large hyphal network, the contribution of AMF to nitrogen uptake in mycorrhizal plants exceeds that of the plant’s own N absorption, with the extracellular mycelium (ERM) pathway accounting for over 42% of N uptake (Calabrese et al., 2016). In addition, AMF mediate the absorption of NO_3_^−^ by regulating the transcription of NRTs, and AMF-induced NO_3_^−^ transporters have been found in many plants (Rui et al., 2022). In this study, genes from the NRT family were significantly upregulated following *C. etunicatum* colonization (**Table 2**). The core gene groups mainly included the peptide transporter family (NPF (NRT/PTR)) and nitrate transporter 2 (NRT2), which are the main nitrogen transporters in plants. Nitrate transporters play crucial roles in the transport of NO_3_^−^ via the ERM pathway and serve as two-way channel proteins for NO_3_^−^ exchange between AMF and plants at the symbiotic interface (Tian et al., 2010). Unlike P and N, there was no clear result for K absorption associated with AMF colonization. However, positive results indicate that AMF colonization may provide specific adaptation mechanisms for host plants to tolerate long-term K deprivation (Calabrese et al., 2016). In this study, the content of K in the roots of *C. etunicatum* significantly increased after colonization, and the content of K in the shoots slightly increased. Numerous studies have shown that AMF can improve potassium absorption in plants by inducing the expression of K^+^ transport systems, which are thought to be involved in phloem loading/unloading, xylem release, and Na^+^/K^+^ homeostasis (Garcia and Zimmermann, 2014). Therefore, we hypothesize that the colonization of *C. etunicatum* might mediate the loading/unloading of arsenic in *P. vittata* phloem through K^+^ transport systems.

In addition to biomass, the content of heavy metals in plants is key to determining the efficiency of phytoremediation. The arsenic content in the shoots and roots of *P. vittata* significantly increased by 1.22- and 1.66-fold, respectively, after *C. etunicatum* inoculation (**Figure 2**). Similarly, the *P. vittata* arsenic EXT increased by 2.74-fold after inoculation with *C. etunicatum*, the BAF increased by 1.33-fold, and the ETF increased by 1.56-fold (**Figure 3**). Therefore, *C. etunicatum* inoculation can improve the rate of *P. vittata* arsenic removal, which is closely related to the increase in plant biomass and aboveground arsenic concentration.

### 4.2 Mechanisms of arsenic uptake and transport by *P. vittata* mediated by *C. etunicatum*

After mycorrhizal colonization, plant roots absorb elements through two pathways: directly through the root epidermis and root hairs (direct pathway) and indirectly through AMF hyphae, which transfer nutrients from external mycorrhizal hyphae to root cortex cells. Our previous research has shown that the ERM pathway provides nearly one-third of the arsenic for AMF-*P. vittata* symbiosis (Pan et al., 2024). In addition, AMF colonization also increased the arsenic content on the root surface, providing more absorbable arsenic to the roots, which may have led to an increase in the amount of arsenic absorbed directly. At present, the main transporters for arsenic uptake by *P. vittata* are PHTs and MIPs. Proteomics has found that the expression levels are the highest in the MIP, P-ATPase, MFS, and ABC families and respond to arsenic treatment, indicating that these families play a key role in arsenic compartmentalization (Yan et al., 2019). However, under low concentrations of arsenic, most ABC transporter genes are downregulated in roots, and most of these gene transcripts are homologous sequences of *OsABCC1*/*AtABCC1*, which are responsible for transporting As(III) into vacuoles in rice and *Arabidopsis* (Leslie et al., 2004; Sun et al., 2023). N and P belong to the same family of elements and have similar properties. NRT family transporters may also have arsenic transport capacity. In rice, the NRT1/PTF family transporter osPTR7 shows DMA transport capacity under arsenic exposure (Tang et al., 2017). PHT1 and PvTIP4;1 are responsible for the uptake of As(V) and As(III), respectively, whereas PvACR2 reduces As(V) to reduce the toxicity of arsenic. In addition, PvACR3, an arsenite antiporter, may be involved in As(III) transport and compartmentalization (Kumar et al., 2016; Zhang et al., 2020; Bai et al., 2023). In this study, the DEGs of the MIP, PHP, NRT, and ACR families were upregulated after *C. etunicatum* colonization, whereas the DEGs of P-ATPase and ABC were downregulated (**Table 2**). Therefore, we speculate that P-ATPase and ABC are responsible for the transport of arsenic to vacuoles in the roots, whereas MIP, PHP, NRT, and ACR are the main transporters for arsenic uptake and transport (PvACR3 is the As(III) efflux transporter). *C. etunicatum* promotes arsenic uptake and efflux in *P. vittata* by increasing the expression of MIP, PHP, and NRT, downregulating the expression of P-ATPase, ABC, and MFS, and inhibiting vacuolar transport (**Figure 8J**).

In addition to confirming arsenic transport *in vitro*, we identified several genes at the leading edge of the GSEA that have not yet been confirmed in *P. vittata* but may be involved in arsenic transport in other plants (**Table 3**). For example, in *Arabidopsis*, *AtTIP1;1* plays an important role in intracellular material transport through vesicle transport mechanisms (Schüssler et al., 2008). In this study, *PvPIP1-1* was identified as the core gene of the MIP gene set, indicating that *C. etunicatum* may also promote the absorption and transport of arsenic by affecting vesicle transport mechanisms. Additionally, *OsPIP2;4*, *OsPIP2;6*, and *OsPIP2;7* have been shown to increase arsenate tolerance and increase biomass accumulation (Mosa et al., 2012). In this study, several *PvPIP2-1* genes were identified as core genes of the MIP gene set, with their expression downregulated following *C. etunicatum* inoculation. Although *PvPIP2;1* has not been found to play a role in arsenite absorption, as a homologous gene of *PvPIP2;4*, it is significant for water absorption and root growth, suggesting that it may also be involved in intracellular arsenic transport (Liu et al., 2020; Israel et al., 2022). In addition, (Xu et al. (2015) reported that *AtNIP3;1* is expressed mainly in the roots and is involved in the absorption of arsenic by *Arabidopsis thaliana*, as well as its transport from roots to shoots. The homologous gene *NIP6;1* has been shown to participate in xylem–phloem boric acid transport (Sun et al., 2018). In this study, *PvNIP 3-1* and *PvNIP 6-1* were also identified as leading edge in the MIP family, with *PvNIP 3-1* being the top DEGs. We hypothesize that these proteins are key arsenite-loading proteins in the xylem and phloem of *P. vittata*.

### 4.3 *C. etunicatum* mediates the rapid reduction and translocation of arsenic in roots

Compared with nonhyperaccumulating plants, *P. vittata*, a hyperaccumulating plant for arsenic, can efficiently transfer arsenic from its roots to its shoots and concentrate it in its leaves, thereby reducing arsenic toxicity to the roots. To facilitate this efficient transport, As(V) is reduced to As(III) in the roots, as As(III) is preferentially excreted from the rhizome cells and loaded into the xylem, allowing it to be transported from the roots to the aboveground parts (Wang et al., 2010; Lei et al., 2012). After colonization with *C. etunicatum*, the As(V)/As(III) ratio in the shoots significantly increased by 1.41-fold, while no significant change was observed in the roots (**Figure 4**). We speculate that this is the result of As(III) being transferred to the shore (ETF increased by 1.56 times). In addition, the arsenate reductase activity of *P. vittata* roots significantly increased by 1.85-fold (**Figure 6**), along with a notable rise in the expression levels of arsenic reduction-related genes (**Figure 9**). These results indicate that after the colonization of *C. etunicatum*, arsenic undergoes a more rapid reduction and translocation toward the roots.

At present, there are two main pathways for arsenic reduction by *P. vittata*: direct reduction to As(III) through the arsenate reductase PvACR2 and the vesicle reduction pathway related to glutathione metabolism (Ellis et al., 2006; Cai et al., 2019). In this study, the expression levels of *PvACR2* and *PvGSTF1* were high, whereas the expression levels of *PvHAC1*, *PvHAC2*, and *Pv4-8* were low. Although previous findings have shown that PvHAC1, PvHAC2, and Pv4-8 all have the ability to reduce As(V), we speculate that they are not the main arsenate reductases of *P. vittata*. GSH is a key substance in the vesicle reduction pathway. The important mechanism for arsenic detoxification in nonhyperaccumulating plants such as *Arabidopsis* and rice is the binding of GSH to arsenite through glutathione S-transferase (GST) (Lei et al., 2012). However, a large amount of As-GSH is present in the leaves and vascular bundles of *P. vittata*. Therefore, the chelation of arsenite with GSH and its transfer to aboveground parts, followed by degradation in feather leaf vacuoles, may be one of the arsenic tolerance mechanisms of *P. vittata* (Schüssler et al., 2008; Tang et al., 2017).

Sun et al. (2023) analyzed the transcriptome of *P. vittata* treated with different arsenic concentrations and found that PvOCT4 and PvGSTF1 were detected only under high arsenic stress. Interestingly, the significant induction of GST expression in nonhyperaccumulator plants resulted in a significant increase in 19 GST transcripts, whereas only three GST transcripts were found in *P. vittata*. Therefore, we speculate that the specific *P. vittata* reduction mechanism under high-arsenic stress is difficult to employ and that a vesicular arsenic transformation mechanism similar to that of nonhyperaccumulators is activated. In this study, although the expression of PvGAPC1 and PvGSTF1 was upregulated after *C. etunicatum* colonization (**Figure 9**), the glutathione metabolism pathway was downregulated (glutathione reduction and NADP+ reduction); this may be because the PvGSTF1 reduction pathway in roots was enhanced after *C. etunicatum* colonization, but arsenic was inhibited in the vacuolar compartment of roots by regulating the glutathione metabolism pathway. Since ABC transporters can store arsenic thiol compounds in vacuoles and *C. etunicatum* colonization downregulates the overall expression of the ABC transporter family, As(III)-GSH in roots undergoes shoot translocation (Lei et al., 2013; Vandana et al., 2020).

### 4.4 Rapid reduction and translocation of arsenic alleviate the arsenic resistance pathway

ROS metabolism is the main physiological pathway for plant arsenic resistance. SOD/CAT and the ascending glutamate cycle are two pathways for clearing ROS (Foyer and Noctor, 2011). Acid and aldehyde metabolism (map00053) are the main metabolic pathways of the SOD/CAT pathway, and we found that the FPKM of l-ascorbate peroxidase 2 (APX, Gene ID: TRINITYDN8544_c0ug1, NCBI: XP_019250347.1) accounts for 30% of this pathway. Previous studies have found that under arsenic treatment, the activities of APX, CAT, and SOD in *P. vittata* roots significantly increase (Campos et al., 2015). The GSEA results of the entire metabolic pathway indicate that this pathway is downregulated after *C. etunicatum* colonization, which may be due to arsenic not being concentrated in the roots as the production of ROS is reduced (Wang et al., 2022). The degradation of misfolded proteins and the GSH metabolism pathway are two important arsenic resistance pathways in *P. vittata* (Yan et al., 2019). In this study, GSEA revealed that the ubiquitin-mediated proteolysis pathway (map04120) gene set was not significantly activated or inhibited after *C. etunicatum* colonization, but protein processing in the endoplasmic reticulum pathway (map04141) and protein pathway (03050) were significantly downregulated (**Figure 9**). Similarly, the glutathione metabolic pathway (map00480) was also downregulated. These results indicate that the arsenic resistance pathway in *P. vittata* roots is alleviated after *C. etunicatum* colonization.

AMF can reduce the biological toxicity of heavy metals by regulating their chemical forms (Li H. et al., 2020). In this study, *C. etunicatum* colonization significantly increased the amount of alcohol-soluble arsenic in the roots and shoots of *P. vittata* and reduced the amount of residual arsenic (**Figure 4**). Arsenic exposure mainly affects the metabolic pathways involved in monoterpenoid biosynthesis, arachidonic acid metabolism, and sesquiterpenoid and triterpenoid biosynthesis in positive mode (Han et al., 2024). According to the KEGG hyperaccumulation results, both the differentially expressed DEGs and the specifically expressed DEGs were strongly enriched in diterpenoid biosynthesis; stilbenoid, diarylheptanoid, and gingerol biosynthesis; and phenylpropanoid biosynthesis (**Figure 6**). Therefore, the colonization of *C. etunicatum* may affect the chemical form of arsenic by promoting the biosynthesis of secondary metabolites in the roots of *P. vittata*. This transformation not only reduces arsenic’s biological toxicity but also enhances its mobility, thereby alleviating the stress associated with arsenic resistance, including the misfolded protein degradation pathways and GSH metabolism pathways.

## 5 Conclusions

Our research results indicate that *C. etunicatum* colonization can increase *P. vittata’s* tolerance to and enrichment of As through the following pathways. First, *C. etunicatum* promotes arsenic uptake by upregulating the expression of the MIP, PHT, and NRT transporter families. Simultaneously, it enhances the activity of arsenate reductase, rapidly reducing arsenic levels in the cytoplasm and vesicles. The expression of the ABC and P-type ATPase protein families is subsequently downregulated to prevent arsenic concentration in the roots and promote its translocation to the leaves. This rapid reduction and upward transposition mechanism of arsenic alleviates the anti-arsenic stress effects on misfolded protein degradation pathways and GSH metabolism pathways, thereby enhancing *P. vittata’s* arsenic tolerance and enrichment ability.

### 5.1 Environmental implications

Arsenic is carcinogenic. *P. vittata* is a plant with high arsenic accumulation. We introduced AMF to improve the efficiency of *P. vittata* arsenic uptake. Under arsenic stress, *C. etunicatum* endows *P. vittata* with greater arsenic resistance and hyperaccumulation ability by mediating the absorption and rapid reduction of arsenic in *P. vittata* roots. After *C. etunicatum* colonization, the arsenic accumulation in *P. vittata* increased by 2.69%–3.36%. These results are helpful for understanding and applying the regulatory mechanism of AMF in plants.

## Data Availability

The original data from all the transcriptome libraries were stored in the NCBI Sequence Read Archive (SRA) under accession number PRJNA1118441.
