# Supplementary material for: Claroideglomus etunicatum enhances Pteris vittata L. arsenic resistance and accumulation by mediating the rapid reduction and transport of arsenic in roots
Source: Front Plant Sci. 2024 Nov 13;15:1464547. doi: 10.3389/fpls.2024.1464547 (PMC11598345; doi:10.3389/fpls.2024.1464547)
Supplement: Supplementary file 1 [file DataSheet1.docx]

**Supporting Information**

***Claroidoglomus etunicatum* enhances *Pteris vittata* L. arsenic resistance and accumulation by mediating the rapid reduction and transport of arsenic in roots**

*Guofei Pan ^a1^, YueZhen Xu ^a1^, WeiZhen Li ^a1^, Linyan Zan ^a^, Xueli Wang ^a,^ **

^a^ Guangxi Key Laboratory for Agro-Environment and Agro-Products Safety, State Key Laboratory for Conservation and Utilization of Subtropical Agri–Bioresources, National Demonstration Center for Experimental Plant Science Education, College of Agriculture, Guangxi University, Nanning 530004, China.

^1^These authors contributed to the work equllly.

**^*^Corresponding author:**

Xueli Wang, E-mail: [wxl0524@126.com](mailto:wxl0524@126.com)

**
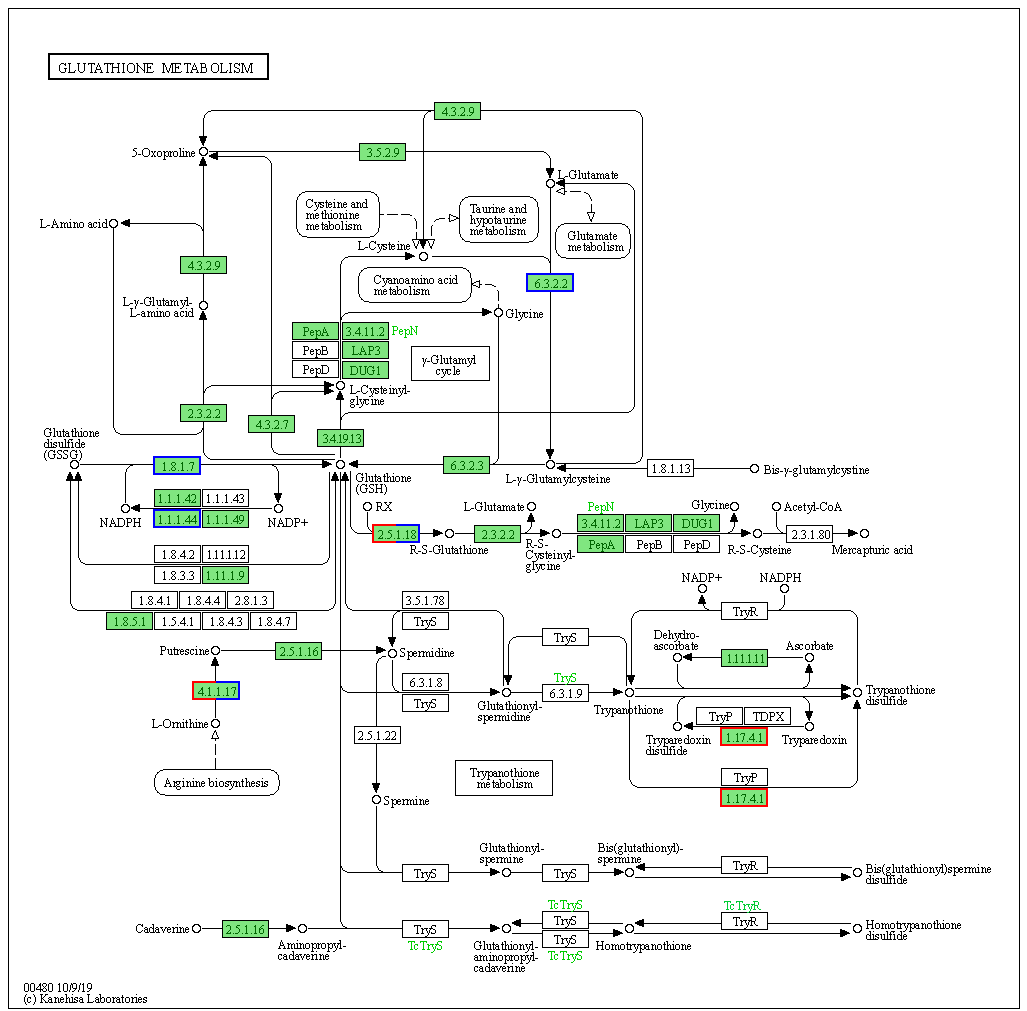
**

**Fig. S1 Differential gene metabolic pathway diagram**

**
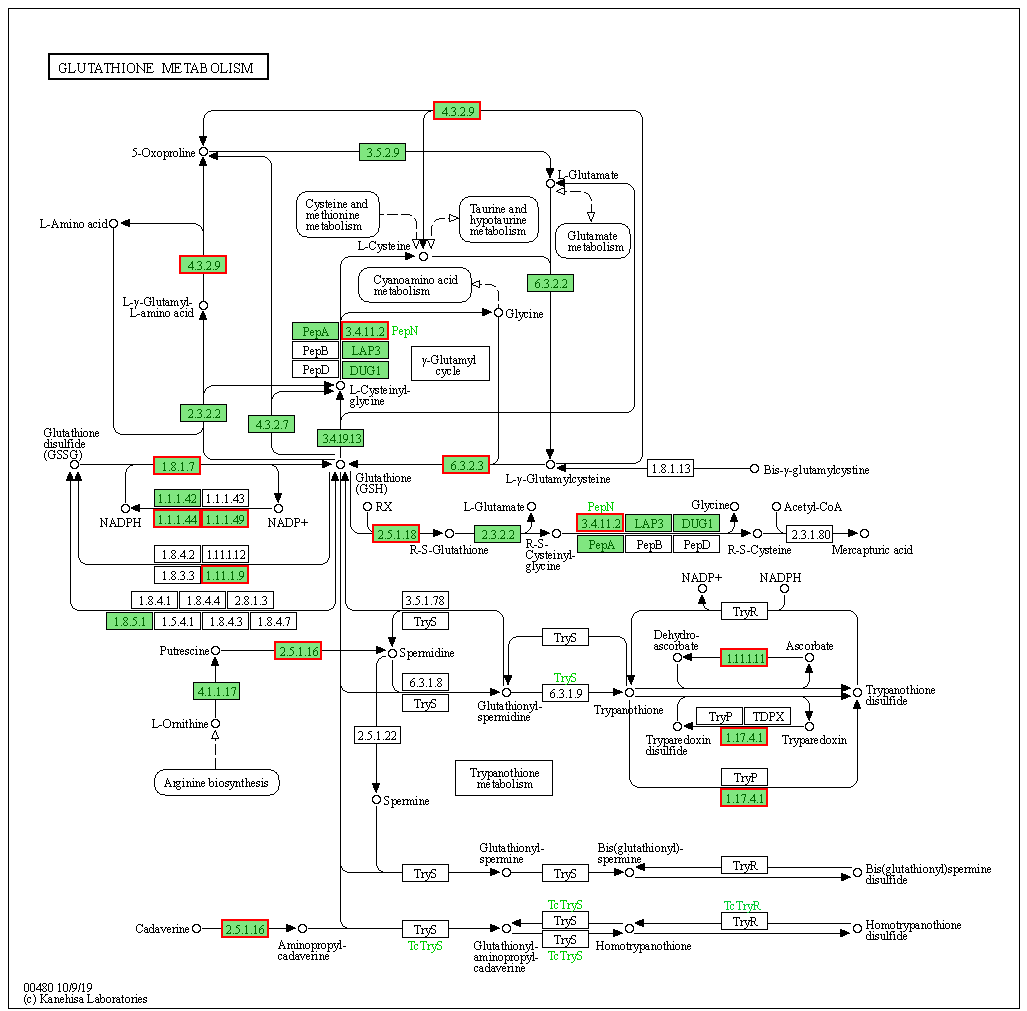
**

**Fig. S2** **Leading Edge metabolic pathway diagram**

**Table S1**

Physical and chemical properties of the tested soil.

| **longitude** | **latitude** | **Total Arsenic (mg kg^-1^)** | **pH** | **Organic matter**  **(g kg^-1^)** | **Available phosphorus**  **(mg kg^-1^)** | **Available potassium**  **(mg kg^-1^)** |
| --- | --- | --- | --- | --- | --- | --- |
| 108.8242 N | 22.7143 E | 7.78 | 4.49 | 12.02 | 1.77 | 38.77 |

**Table S2**

Primers of protein gene were determined

| **Gene Name** | **Gene ID** | **Primer-F** | **Primer-R** |
| --- | --- | --- | --- |
| *β-Actin* |  | GGGCAGTATTTCCAAGCATAGTGGG | TGCCTCGCTTTGATTGAGCCTCATC |
| *PvPIP1-1* | TRINITY_DN1610_c1_g1 | GCTTGGTGCCGAGATTGTTG | TGGTCATCCCATGCTTGCTT |
| *PvNIP6-1* | TRINITY_DN13097_c0_g1 | GGAGGGTGCTGCGATTATGA | AAAGGCACCTCTGACCATCG |
| *PvPIP2-1* | TRINITY_DN1669_c1_g1 | GCCACCCTCCTCTTCCTCTA | ATTGCGCCACCATGTACAGA |
| *PvPht1;1* | TRINITY_DN35649_c0_g1 | CCCTCATTGCACTCTGCTCA | CAACCGTGCAGGGAAGATCT |
| *PvPht2;1* | TRINITY_DN11245_c0_g1 | CATGGAGCCGGTGAAGTAGG | TTGGCAAGCTTGACTCCCAT |
| *PvGSTF1* | TRINITY_DN26745_c0_g1 | GCCACCCTCCTCTTCCTCTA | ATTGCGCCACCATGTACAGA |
| *PvGAPC1* | TRINITY_DN10105_c0_g1 | GTATGACTCTACCCACGGCG | TCTCGTTGACACCCATCACG |
| *PvACr2* | TRINITY_DN2794_c0_g2 | CCTCACAAAAGGCTTCCCCA | AAGCCGCACCACAGGTTTAT |
| *PvOCT4* | TRINITY_DN9350_c0_g1 | GTCGTGCTGGTTTGGTTCAC | GCGTCTACCGATATGCCGAA |
| *PvPht1;3* | TRINITY_DN688_c0_g1 | AAGCCGATACAGATGCAGGG | CCCCACCTGATTCTTGTCCC |
| *PvNRT1/ PTR FAMILY 5.1* | TRINITY_DN158_c0_g1 | CAACAAGCATTCCACGGGTG | CCCCATCTGCGTCGTTAAGT |
| *PvNRT1/ PTR FAMILY 6-3* | TRINITY_DN13756_c0_g1 | ACACCCACCAACTCTCGTTC | CTCCACTGAGCAAGTCCTCC |
| *PvNRT1/ PTR FAMILY 6-3* | TRINITY_DN14280_c0_g1 | ATGAGCAAGGGGTGTGGATG | AGCTGGTGGGATCTTGAAGC |
| *PvPATP1* | TRINITY_DN10629_c0_g1 | CCTGGTGATGTTGTCGAGCT | ACTGCCATACTCTCTCCGGT |
| *PvPATP1-3* | TRINITY_DN2110_c0_g1 | GCCTTCAACACGGGAGATGA | GCGCCATACTTTGACACAGC |
| *PvPATP2* | TRINITY_DN7892_c0_g1 | GTGAAGCCCAGTCCCTTACC | GTGAGCCGACTTTGACTCCA |

**Table S3**

Sequencing data statistics table

| **Sample** | **Raw reads** | **Raw bases** | **Clean reads** | **Clean bases** | **Error rate(%)** | **Q20(%)** | **Q30(%)** | **GC content(%)** |
| --- | --- | --- | --- | --- | --- | --- | --- | --- |
| CE4 | 54163030 | 8178617530 | 53459024 | 7954592963 | 0.0255 | 97.82 | 93.73 | 47.54 |
| CE2 | 58907230 | 8894991730 | 58255562 | 8685506981 | 0.025 | 98 | 94.17 | 47.41 |
| CE1 | 46849630 | 7074294130 | 46221764 | 6879119225 | 0.0253 | 97.85 | 93.84 | 47.89 |
| CK3 | 47776730 | 7214286230 | 47185188 | 7011220057 | 0.0252 | 97.91 | 93.93 | 47.74 |
| CK2 | 49030528 | 7.4E+09 | 48424306 | 7.22E+09 | 0.0253 | 97.87 | 93.83 | 47.43 |
| CK1 | 55384560 | 8.36E+09 | 54719576 | 8.16E+09 | 0.0251 | 97.99 | 94.11 | 47.68 |
